# Optimization of Evidence-Based Heart Failure Medications After an Acute Heart Failure Admission: A Secondary Analysis of the STRONG-HF Randomized Clinical Trial

**DOI:** 10.1001/jamacardio.2023.4553

**Published:** 2023-12-27

**Authors:** Gad Cotter, Benjamin Deniau, Beth Davison, Christopher Edwards, Marianna Adamo, Mattia Arrigo, Marianela Barros, Jan Biegus, Jelena Celutkiene, Kamilė Čerlinskaitė-Bajorė, Ovidiu Chioncel, Alain Cohen-Solal, Albertino Damasceno, Rafael Diaz, Gerasimos Filippatos, Etienne Gayat, Antoine Kimmoun, Carolyn S.P. Lam, Marco Metra, Maria Novosadova, Peter S. Pang, Matteo Pagnesi, Piotr Ponikowski, Hadiza Saidu, Karen Sliwa, Koji Takagi, Jozine M. Ter Maaten, Daniela Tomasoni, Adriaan Voors, Alexandre Mebazaa

**Affiliations:** 1 Université Paris Cité, INSERM UMR-S 942(MASCOT), Paris, France; 2 Momentum Research Inc, Durham, North Carolina; 3 Heart Initiative, Durham, North Carolina; 4 Department of Anesthesiology and Critical Care and Burn Unit, Saint-Louis and Lariboisière Hospitals, FHU PROMICE, DMU Parabol, APHP.Nord, Paris, France; 5 Cardiology, ASST Spedali Civili and Department of Medical and Surgical Specialties, Radiological Sciences, and Public Health, University of Brescia, Brescia, Italy; 6 Department of Internal Medicine, Stadtspital Zurich, Zurich, Switzerland; 7 Institute of Heart Diseases, Wroclaw Medical University, Wrocław, Poland; 8 Clinic of Cardiac and Vascular Diseases, Institute of Clinical Medicine, Faculty of Medicine, Vilnius University, Vilnius, Lithuania; 9 Emergency Institute for Cardiovascular Diseases “Prof. C.C.Iliescu,” University of Medicine “Carol Davila,” Bucharest, Romania; 10 Department of Cardiology, APHP Nord, Lariboisière University Hospital, Paris, France; 11 Faculty of Medicine, Eduardo Mondlane University, Maputo, Mozambique; 12 Estudios Clínicos Latinoamérica, Instituto Cardiovascular de Rosario, Rosario, Argentina; 13 National and Kapodistrian University of Athens, School of Medicine, Attikon University Hospital, Athens, Greece; 14 Université de Lorraine, Nancy; INSERM, Défaillance Circulatoire Aigue et Chronique; Service de Médecine Intensive et Réanimation Brabois, CHRU de Nancy, 54511 Vandoeuvre-lès-Nancy, France; 15 National Heart Centre Singapore and Duke-National University of Singapore, Singapore; 16 Department of Cardiology, University of Groningen, University Medical Centre Groningen, Groningen, Netherlands; 17 Department of Emergency Medicine, Department of Medicine, Indiana University School of Medicine, Indianapolis; 18 Murtala Muhammed Specialist Hospital / Bayero University Kano, Kano, Nigeria; 19 Cape Heart Institute, Department of Medicine and Cardiology, Groote Schuur Hospital, University of Cape Town, Cape Town, South Africa

## Abstract

**Importance:**

The Safety, Tolerability, and Efficacy of Rapid Optimization, Helped by N-Terminal Pro–Brain Natriuretic Peptide Testing of Heart Failure Therapies (STRONG-HF) trial strived for rapid uptitration aiming to reach 100% optimal doses of guideline-directed medical therapy (GDMT) within 2 weeks after discharge from an acute heart failure (AHF) admission.

**Objective:**

To assess the association between degree of GDMT doses achieved in high-intensity care and outcomes.

**Design, Setting, and Participants:**

This was a post hoc secondary analysis of the STRONG-HF randomized clinical trial, conducted from May 2018 to September 2022. Included in the study were patients with AHF who were not treated with optimal doses of GDMT before and after discharge from an AHF admission. Data were analyzed from January to October 2023.

**Interventions:**

The mean percentage of the doses of 3 classes of HF medications (renin-angiotensin system inhibitors, β-blockers, and mineralocorticoid receptor antagonists) relative to their optimal doses was computed. Patients were classified into 3 dose categories: low (<50%), medium (≥50% to <90%), and high (≥90%). Dose and dose group were included as a time-dependent covariate in Cox regression models, which were used to test whether outcomes differed by dose.

**Main Outcome Measures:**

Post hoc secondary analyses of postdischarge 180-day HF readmission or death and 90-day change in quality of life.

**Results:**

A total of 515 patients (mean [SD] age, 62.7 [13.4] years; 311 male [60.4%]) assigned high-intensity care were included in this analysis. At 2 weeks, 39 patients (7.6%) achieved low doses, 254 patients (49.3%) achieved medium doses, and 222 patients (43.1%) achieved high doses. Patients with lower blood pressure and more congestion were less likely to be uptitrated to optimal GDMT doses at week 2. As a continuous time-dependent covariate, an increase of 10% in the average percentage optimal dose was associated with a reduction in 180-day HF readmission or all-cause death (primary end point: adjusted hazard ratio [aHR], 0.89; 95% CI, 0.81-0.98; *P* = .01) and a decrease in 180-day all-cause mortality (aHR, 0.84; 95% CI, 0.73-0.95; *P* = .007). Quality of life at 90 days, measured by the EQ-5D visual analog scale, improved more in patients treated with higher doses of GDMT (mean difference, 0.10; 95% CI, −4.88 to 5.07 and 3.13; 95% CI, −1.98 to 8.24 points in the medium- and high-dose groups relative to the low-dose group, respectively; *P* = .07). Adverse events to day 90 occurred less frequently in participants with HIC who were prescribed higher GDMT doses at week 2.

**Conclusions and Relevance:**

Results of this post hoc analysis of the STRONG-HF randomized clinical trial show that, among patients randomly assigned to high-intensity care, achieving higher doses of HF GDMT 2 weeks after discharge was feasible and safe in most patients.

**Trial Registration:**

ClinicalTrials.gov Identifier: NCT03412201

## Key Points

**Question:**

What variables are associated with success in uptitration of guideline-directed medical therapy (GDMT) after discharge from a hospital admission for acute heart failure (AHF)?

**Findings:**

In this secondary analysis of the Safety, Tolerability, and Efficacy of Rapid Optimization, Helped by N-Terminal Pro–Brain Natriuretic Peptide Testing of Heart Failure Therapies (STRONG-HF) randomized clinical trial including 515 patients, 2 weeks after discharge, medium- to high-dose GDMT was prescribed in more than 90% of patients. Patients with lower blood pressure, more congestion, and characteristics denoting higher risk were less uptitrated; patients prescribed more GDMT had lower rates of readmission for HF or death through 6 months and more improved quality of life.

**Meaning:**

When patients with AHF can tolerate higher doses of GDMT, results suggest that all efforts should be made to rapidly uptitrate to optimal doses of the 3 and (likely) 4 pillars of HF medications, including renin-angiotensin receptor system inhibitors, β-blockers, mineralocorticoid receptor antagonists, and sodium-glucose transport protein 2 inhibitors.

## Teaser

This secondary analysis of the Safety, Tolerability, and Efficacy of Rapid Optimization, Helped by N-Terminal Pro–Brain Natriuretic Peptide Testing of Heart Failure Therapies (STRONG-HF) randomized clinical trial investigates what variables are associated with success in uptitration of guideline-directed medical therapy after hospital discharge for an acute heart failure admission.

## Introduction

Acute heart failure (AHF) is associated with a high rate of readmission and death.^1,2,3^ Recently, the Safety, Tolerability, and Efficacy of Rapid Optimization, Helped by N-Terminal Pro–Brain Natriuretic Peptide (NT-proBNP) Testing of Heart Failure Therapies (STRONG-HF) trial showed that an intensive strategy of rapid uptitration of guideline-directed medical therapy (GDMT) combined with close follow-up after an AHF admission was safe, reduced the risk of 180-day all-cause death or heart failure (HF) readmission, and improved quality of life compared with usual care,^4^ confirming previous observational studies.^5,6,7,8,9^ However, in the STRONG-HF trial, despite recommendations for uptitration of GDMT to 100% of maximally recommended doses at week 2, not all patients were prescribed 100% of GDMT at week 2 after randomization and discharge. The aim of this post hoc secondary analysis was to assess the association between the level of uptitration of GDMT achieved and outcomes in the STRONG-HF study.

## Methods

### Study Design

The design and main results of the STRONG-HF trial have been previously described.^4^ Briefly, the STRONG-HF trial was an international, multicenter, open-label, randomized clinical trial designed to compare the safety and efficacy of a high-intensity care (HIC) strategy comprising early uptitration of oral HF medications including β-blockers (BBs), renin-angiotensin receptor system inhibitors (RASis), and mineralocorticoid receptor antagonists (MRAs) vs usual care (UC) in 1078 patients admitted to the hospital for AHF. The study was approved by appropriate competent authorities, and all sites obtained approval from ethics committees. All patients provided written informed consent. This study followed the Consolidated Standards of Reporting Trials (CONSORT) reporting guidelines.

### Patient Population

Inclusion criteria in the STRONG-HF trial were admission for AHF within 72 hours before screening, hemodynamic stability with any left ventricular ejection fraction (LVEF), a high NT-proBNP level greater than 1500 pg/mL, and the absence of treatment with optimal doses of oral HF therapies at 1 week before admission, at screening, and just before randomization, which occurred within 2 days before anticipated hospital discharge. To be eligible, patients had been prescribed either (1) one-half or less the optimal dose of RASi, no BB, and one-half or less the optimal dose of MRA or (2) no RASi, one-half or less the optimal dose of BB, and one-half or less the optimal dose of MRA. Eligible patients were randomly assigned 1:1 within strata defined by LVEF (≤40 or >40%) and country as previously described.^4^ Patients self-identified with the following race and ethnicity categories: Black, Native American, Pacific Islander, White, or other race or ethnic group (included Berber, Gypsy, Europiod, and not specified). Race and ethnicity information was collected to characterize the patients enrolled.

### Intervention

Patients assigned to HIC had follow-up visits at 1, 2, 3, and 6 weeks after, with a subsequent study visit at day 90. BB, RASi, and MRA medications were uptitrated to one-half the optimal doses at randomization and to full optimal doses at week 2 as long as indications were that uptitration was safe. Per protocol, investigators were asked to increase diuretics if the patient was congested and to not uptitrate BBs and increase diuretics if the NT-proBNP level was more than 10% higher than the predischarge level. If the systolic blood pressure was lower than 95 mm Hg, serum potassium level was greater than 5.0 mmol/L (to convert potassium to milliequivalents per liter, divide by 1) or estimated glomerular filtration rate (eGFR) was less than 30, RASi and/or MRA medications were not to be uptitrated. If any of the safety indicators required a delay in the uptitration, a safety visit including all assessments was required 1 week after any uptitration. Patients in the UC group were followed up after discharge according to the local practice and were evaluated again by the study team at day 90 after randomization. Finally, patients in both groups were contacted at day 180 to assess the occurrence of rehospitalizations and death.

### Study End Points

The study’s primary end point was the composite of first HF rehospitalization or all-cause death at day 180. Secondary end points were change in the EQ-5D visual analog scale (EQ-VAS) score^10^ from baseline to day 90, 180-day all-cause death, and the composite of first HF rehospitalization or all-cause death at day 90.

### Statistical Analysis

To provide a measure of the degree of full GDMT implementation, the mean percentage of the doses of the 3 classes of HF medications (RASi, BB, and MRA) relative to their optimal doses was computed. For instance, a patient with HF in the HIC arm receiving 30% of the optimal dose of RASi, 40% of the optimal dose of BB, and 50% of the optimal dose of MRA at week 2 after discharge would have an average percentage optimal dose of HF medications of (30 + 40 + 50) / 3 = 40%. The average percentage of optimal doses was divided into 3 categories: low (<50%), medium (≥50% to <90%), and high (≥90%).

Continuous variables are presented as mean and SD, adjusted (least-squares) mean and associated SE, or, in the case of log-transformed variables, as geometric mean and 95% CI. Categorical variables are presented as absolute and relative frequency. Dose categories were compared using the Jonckheere trend test for continuous variables, Cochron-Armitage trend test for binary variables, Cochran-Mantel-Haenszel (CMH) test of general association for categorical variables, and CMH test of nonzero correlation for ordinal variables.

Changes in vital signs and laboratory parameters were compared between dose groups using analysis of covariance (ANCOVA) adjusted for either the baseline or the week 2 value, respectively. ANCOVA with adjustment for baseline EQ-VAS and randomization stratification factors (LVEF ≤40 or >40% and region) was used to compare differences with respect to change in EQ-VAS; patients enrolled in Mozambique were excluded from these analyses because the EQ-5D translation in that country was not linguistically validated.

We examined the association of the average percentage optimal dose with outcomes in 2 ways. First, we used Cox regression treating the average percentage optimal dose—in dose categories and separately as a continuous variable—as a time-dependent covariate, thus attributing the follow-up during which a patient was prescribed a particular dose level to the time at risk for that dose. Kaplan-Meier curves for the time-dependent dose group covariate were plotted using the method of Snapinn, Jiang, and Iglewicz,^11^ an extension of the method by Simon and Makuch.^12^ Second, we used landmark analysis^13^ in which patients in the HIC group were classified by the average percentage optimal dose at week 2 (when full uptitration was to be achieved) and excluding any patients who had experienced the event of interest before week 2. We also examined the change in hazard for each outcome as a function of average percentage optimal dose at week 2 as a continuous variable, modeled as a restricted cubic spline with 3 knots, using Cox regression. Because few patients were prescribed an average of less than 50% of optimal doses, we repeated these analyses including patients in the UC group assuming that their oral HF medication doses did not change between randomization and day 90. Covariates for adjustment were selected from factors shown to be prognostic in previous studies^14,15,16,17^ using backward selection in the UC group with a criterion for staying of *P* < .10 across 10 multiple imputation data sets. Analyses of day 180 end points excluded patients at sites that did not enroll patients under the amended protocol, which allowed patient follow-up through day 180. Additionally, analyses of day 180 end points down-weighted results proportional to one-half its sample size for patients enrolled before an amendment where the primary end point was changed from 90 to 180 days.

A multivariable linear regression model predicting the average percentage optimal dose at week 2 as a continuous variable was constructed in the HIC group. Ten multiple imputation data sets were used to handle missing covariates. Variables with a nonlinear association with the outcome were transformed, and backward selection retained variables with *P* < .10 across the imputation data sets in the multivariable model.

The occurrence of adverse events based on the average percentage optimal dose of HF medications categories at week 2 in the HIC group were examined. Events that occurred from week 2 through study day 90 were included in these analyses. An examination of the trend across the 3 dose categories was conducted for select system organ classes and preferred terms.

Statistical analyses were performed using SAS, version 9.4 (SAS Institute) and R, version 4.2.3 (R Core Team).^18^ Two-sided *P* values <.05 were considered statistically significant. Data were analyzed January to October 2023.

## Results

### Study Population

**Figure 1.**
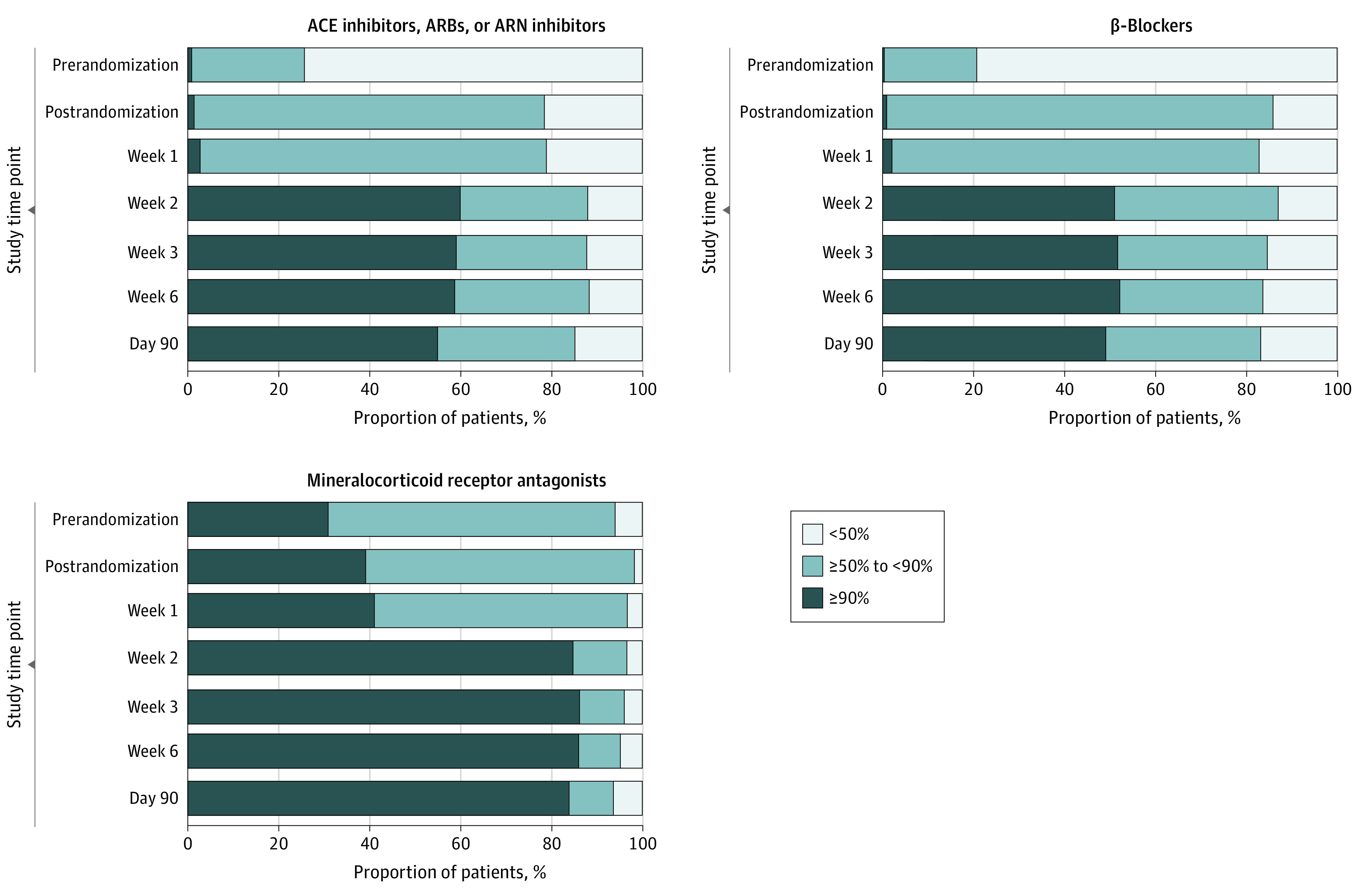
Change in Doses of Guideline-Directed Medical Therapy in the High-Intensity Arm by Week to Day 90 ACE indicates angiotensin-converting enzyme; ARB, angiotensin receptor blocker; ARN, angiotensin receptor-neprilysin inhibitor.

Among the 1078 patients included in the STRONG-HF trial, 515 (mean [SD] age, 62.7 [13.4] years; 311 male [60.4%]; 204 female [39.6%]) of 542 patients randomly assigned to the HIC group had week 2 medication data available. Patients self-identified with the following race and ethnicity categories: 113 Black (21.9%), 1 Native American (0.4%), 1 Pacific Islander (0.4%), 393 White (76.3%), and 7 other race or ethnic group (1.4%). A final follow-up visit at day 180 was not completed for the following: (1) 32 of 515 patients (6.2%) in the HIC group because the site did not follow patients to day 180; (2) 29 of 515 patients (5.6%) because the patient died before day 180; (3) 23 of 515 patients (4.4%) because the study was prematurely terminated; and 5 of 515 patients (1.0%) because of other reasons. In the usual care group a final follow-up visit at day 180 was not completed for 34 of 535 [6.4%] patients because the site did not follow patients to day 180, 48 of 534 [9.0%] because the patient died prior to day 180, 23 of 534 [4.3%] because the study was prematurely terminated and 4 of 534 [0.7%] because of other reasons. At week 2 after randomization, not all patients in the HIC group were fully uptitrated to 100% of maximally recommended GDMT doses, as specified by the protocol; 39 patients (7.6%) were prescribed, on average, less than 50%, 254 patients (49.3%) were prescribed 50% to 90%, and 222 patients (43.1%) were prescribed 90% or more of maximum recommended GDMT doses. The baseline characteristics of patients according to prescribed GDMT at week 2 are presented in Table 1. In the HIC arm, 27 of 542 patients (5.0%) were missing some medication data at the week 2 visit. The GDMT prescription uptitration by medication type and visit in the HIC arm is depicted in Figure 1. Of the recommended doses of RASi medication at week 2 in the HIC arm, of 515 patients, 62 (12.0%) were prescribed less than 50%, 155 (28.0%) were prescribed 50% to 90%, and 309 (60%) were prescribed greater than 90%. The proportion of 515 patients being prescribed less than 50%, 50% to 90%, and greater than 90% of recommended doses of BB were 67 (13%), 185 (35.9%), and 263 (51.1%), respectively, and for MRA, the proportions were 18 (3.5%), 61 (11.8%), and 436 (84.7%), respectively. Patients in the HIC group who were prescribed higher doses of GDMT were younger, more often self-identified with Black race, and were enrolled in non-European countries. History of acute coronary syndrome and atrial fibrillation or atrial flutter was less frequent in patients prescribed higher GDMT doses, whereas history of HF was more frequent in this category. Systolic blood pressure was higher in patients prescribed higher GDMT doses. Baseline EQ-VAS was lower, on average, in patients prescribed higher GDMT doses. Patients prescribed higher GDMT doses at week 2 had also been more frequently prescribed MRAs just before randomization, whereas prerandomization prescription of other medications (eg, RASi, BBs, and loop diuretics) were similar.

**Table 1.** Baseline Characteristics of Patients in High-Intensity Care by Average Percentage Optimal Dose Categories at Week 2

Parameter	Average dose			*P* value^a^
	<50% (n = 39)	≥50-<90% (n = 254)	≥90% (n = 222)	
Age, mean (SD), y	64.3 (12.87)	64.2 (12.53)	60.6 (14.23)	.005
Sex, No. (%)				
Male	25 (64.1)	161 (63.4)	125 (56.3)	.12
Female	14 (35.9)	93 (36.6)	97 (43.7)	
Self-reported race, No. (%)				
Black	6 (15.4)	27 (10.6)	80 (36.0)	<.001
Native American	0	1 (0.4)	0	
Pacific Islander	0	1 (0.4)	0	
White	32 (82.1)	219 (86.2)	142 (64.0)	
Other^b^	1 (2.6)	6 (2.4)	0	
Systolic blood pressure at baseline, mean (SD), mm Hg	118.8 (12.56)	121.1 (10.98)	126.8 (14.86)	<.001
NT-proBNP at screening, geometric mean (95% CI), ng/L	6007.1 (4804.0-7511.5)	6150.8 (5723.5-6609.9)	6091.0 (5675.1-6537.4)	.71
NT-proBNP at baseline, geometric mean (95% CI), ng/L	3514.8 (2780.2-4443.6)	3214.6 (2973.2-3475.7)	3166.8 (2921.2-3433.1)	.59
History of atrial fibrillation or atrial flutter or present at screening, No. (%)	16 (41.0)	123 (48.4)	79 (35.6)	.04
Geographical region, No. (%)				
Europe	31 (79.5)	212 (83.5)	134 (60.4)	<.001
Non-Europe	8 (20.5)	42 (16.5)	88 (39.6)	
Baseline EQ-VAS, mean (SD)	61.4 (15.16)	60.8 (15.35)	56.1 (14.94)	.003
Stroke or transient ischemic attack, No. (%)	6 (15.4)	28 (11.0)	17 (7.7)	.09
Severe liver disease, No. (%)	0	2 (1.0)	1 (0.5)	>.99
Psychiatric or neurologic disorder, No. (%)	0	5 (2.0)	2 (0.9)	>.99
Malignancies, No. (%)	3 (7.7)	7 (2.8)	7 (3.2)	.43
Diabetes, No. (%)	10 (25.6)	76 (29.9)	56 (25.3)	.49
Diabetes control method, No. (%)				
Insulin, No. (%)	6 (15.4)	23 (9.1)	16 (7.2)	.13
Diet only, No. (%)	8 (20.5)	58 (22.8)	30 (13.6)	.03
Oral antidiabetic agents, No. (%)	7 (17.9)	54 (21.3)	39 (17.6)	.54
Pulmonary embolism, No. (%)	1 (2.6)	10 (3.9)	2 (0.9)	.11
Acute coronary syndrome, No. (%)	12 (30.8)	103 (40.6)	42 (18.9)	<.001
Coronary artery bypass surgery, No. (%)	3 (7.7)	17 (6.7)	7 (3.2)	.08
Percutaneous transluminal coronary intervention, No. (%)	5 (12.8)	52 (20.5)	20 (9.0)	.02
Angina Canadian Cardiovascular Society class 2 or higher, No. (%)	3 (7.7)	44 (17.4)	20 (9.0)	.17
Moderate or severe chronic obstructive pulmonary disease or asthma, No. (%)	2 (5.1)	6 (2.4)	2 (0.9)	.07
Cardiac resynchronization therapy, No. (%)	0	2 (0.8)	1 (0.5)	>.99
Automatic internal cardiac defibrillator, No. (%)	0	2 (0.8)	1 (0.5)	>.99
Heart failure history, No. (%)				
History of heart failure	32 (82.1)	212 (83.5)	199 (89.6)	.05
NYHA class 1 mo before hospital admission, No. (%)				
1	2 (5.4)	17 (7.2)	10 (4.8)	.002
2	7 (18.9)	59 (25.1)	69 (32.9)	
3	15 (40.5)	87 (37.0)	105 (50.0)	
4	13 (35.1)	72 (30.6)	26 (12.4)	
Ischemic etiology, No. (%)	22 (56.4)	141 (55.5)	81 (36.7)	<.001
Left ventricular ejection fraction, mean (SD), %	34.4 (11.96)	36.3 (12.91)	37.4 (12.11)	.22
Hospitalized for heart failure in the past year, No. (%)	11 (28.2)	69 (27.2)	53 (23.9)	.39
No. of heart failure hospitalizations in the past year, mean (SD)	0.4 (0.82)	0.4 (0.72)	0.3 (0.51)	.19
History of atrial fibrillation or atrial flutter, No. (%)	17 (43.6)	127 (50.0)	81 (36.5)	.02
Type of atrial fibrillation or atrial flutter, No. (%)				
Paroxysmal	4 (25.0)	26 (20.6)	22 (27.5)	.82
Permanent	9 (56.3)	79 (62.7)	44 (55.0)	
Persistent	3 (18.8)	21 (16.7)	14 (17.5)	
Local laboratory, mean (SD)				
Hemoglobin, g/L	131.1 (22.00)	139.4 (21.10)	134.0 (18.73)	.03
Lymphocytes, %	24.1 (10.11)	26.7 (9.36)	29.1 (10.39)	.004
White blood cells, 10^9^/L	6.9 (2.66)	7.0 (1.80)	6.6 (1.91)	.03
Glucose, mmol/L	6.0 (2.99)	6.4 (2.66)	5.9 (1.92)	.51
Creatinine, μmol/L	123.7 (53.76)	106.4 (28.14)	103.2 (24.34)	.05
Potassium, mmol/L	4.3 (0.56)	4.3 (0.43)	4.2 (0.44)	.002
Sodium, mmol/L	140.3 (4.53)	140.3 (4.32)	139.9 (3.38)	.07
Urea, mmol/L	10.3 (5.50)	8.2 (3.31)	7.5 (3.21)	<.001
ALT, U/L	27.9 (24.04)	31.2 (32.49)	31.2 (67.43)	.07
Total bilirubin, μmol/L	18.4 (12.28)	19.1 (11.43)	15.7 (12.45)	<.001
Total cholesterol, mmol/L	3.8 (1.11)	4.0 (1.06)	4.5 (1.10)	<.001
Oral heart failure medications taken before randomization, No. (%)				
ACE inhibitors/ARBs/ARN inhibitors	24 (61.5)	161 (63.6)	154 (69.4)	.16
β-Blockers	15 (38.5)	91 (36.0)	67 (30.2)	.15
Mineralocorticoid receptor antagonists	34 (87.2)	239 (94.5)	214 (96.4)	.03
Loop diuretic	37 (94.9)	247 (97.6)	211 (95.0)	.40

Abbreviations: ACE, angiotensin-converting enzyme; ALT, alanine aminotransferase; ARB, angiotensin receptor blocker; ARN, angiotensin receptor-neprilysin inhibitor; CMH, Cochran-Mantel-Haenszel; EQ-VAS, EQ-5D visual analog scale; NT-proBNP, N-terminal pro–brain natriuretic peptide; NYHA, New York Heart Association.

SI conversion factor: To convert hemoglobin to grams per deciliter, divide by 10; to convert white blood cell count to per microliter, divide by 0.001; to convert glucose to milligrams per deciliter, divide by 0.0555; to convert creatinine to milligrams per deciliter, divide by 88.4; to convert potassium and sodium to milliequivalents per liter, divide by 1; to convert urea to milligrams per deciliter, divide by 0.167; to convert ALT to microkatals per liter, multiply by 0.0167; to convert bilirubin to milligrams per deciliter, divide by 17.104; to convert total cholesterol to milligrams per deciliter, divide by 0.0259.

^a^ Jonckheere trend test for continuous variables, Cochron-Armitage trend test for binary variables, CMH general association for categorical variables, and CMH nonzero correlation for ordinal variables.

^b^ Other reported races (n = 7) included Berber, Gypsy, Europiod, and not specified.

### Changes in Vital Signs, Signs of Congestion, and Laboratory Values

Changes in vital signs, signs of congestion, and laboratory values are depicted in eTable 1 and eFigure 1 to 10 in Supplement 1. Patients who were uptitrated to higher GDMT doses at week 2 had greater reductions in blood pressure, smaller reduction in weight, and greater reductions in ALT level from week 2 to day 90. Given that the uptitration of GDMT medications at week 2 and beyond depended on certain safety parameters, including NT-proBNP level relative to the discharge level, potassium level, blood pressure, pulse, and kidney function; patients who were prescribed lower GDMT doses at week 2 had larger early changes in these parameters (eFigure 1-10 in Supplement 1).

### Primary Outcome and Mortality by Average Dose of HF GDMT

**Figure 2.**
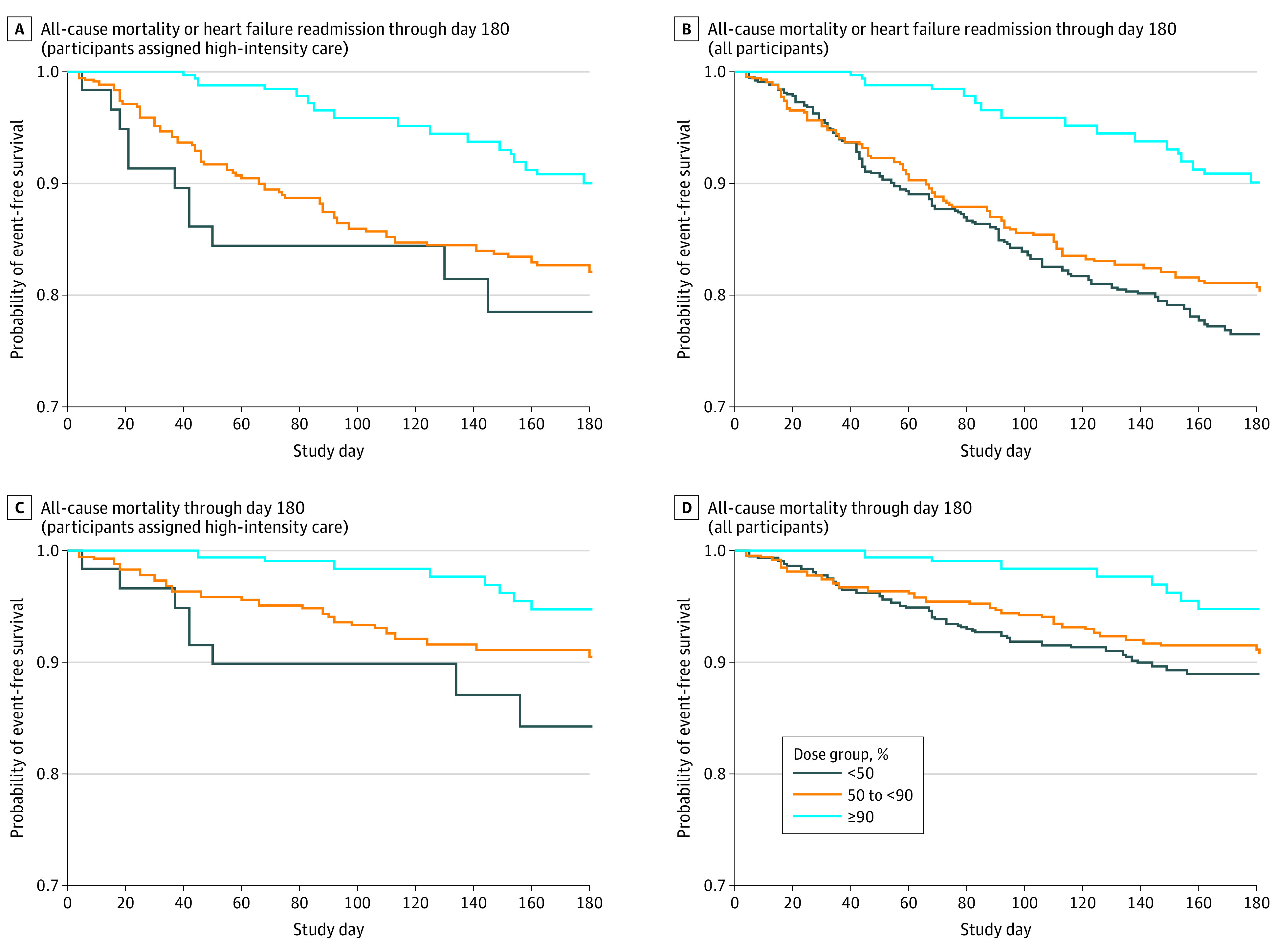
Time-Dependent Kaplan-Meier Curves A, All-cause mortality or heart failure readmission through day 180 (patients assigned high-intensity care). B, All-cause mortality or heart failure readmission through day 180 (all patients). C, All-cause mortality through day 180 (patients assigned high-intensity care). D, All-cause mortality through day 180 (all patients).

Considered as a time-dependent covariate, being in a higher dose category in the HIC group was associated with a lower risk of the primary outcome, 180-day HF readmission or all-cause death, with adjusted hazard ratios (aHRs) of 0.96 (95% CI, 0.46-1.96) and 0.53 (95% CI, 0.23-1.21) for the medium- and high-dose categories relative to the low-dose category, respectively (*P* = .11) (Table 2 and Figure 2A). Including patients in the UC group assuming their GDMT doses did not change between randomization and day 90, the association between GDMT dose category and the primary outcome was strengthened, with aHRs of 0.81 (95% CI, 0.60-1.11) and 0.41 (95% CI, 0.25-0.68) for the medium- and high-dose categories, respectively (*P* = .003) (Table 2 and Figure 2B). As a continuous time-dependent covariate, each increase of 10% in the average percentage optimal dose was associated with a reduction in the risk of 180-day HF readmission or all-cause death with an aHR of 0.89 (95% CI, 0.81-0.98; *P* = .01) in the HIC group only and an aHR of 0.90 (95% CI, 0.85-0.95; *P* <.001) in all participants. These associations were similar when the analysis was done using a landmark at 2 weeks (eTables 2 and 3 and eFigure 11 and 12 in Supplement 1). The association of the average percentage of GDMT optimal dose at week 2 as a continuous variable demonstrates a similar pattern of decreasing hazard of HF readmission or death with increasing HF medication dose (eFigure 15 and 16 in Supplement 1).

**Table 2.** Clinical Outcomes by Average Percentage Optimal Dose as Time Dependent Covariates in Patients Assigned High-Intensity Care and All Patients

End point (patients assigned high-intensity care)	Unadjusted		Adjusted	
	HR (95% CI)	*P* value	HR (95% CI)	*P* value
All-cause death or heart failure readmission by day 180^a^				
Average dose <50%	1 [Reference]	.03	1 [Reference]	.11
Average dose 50-<90%	0.84 (0.41-1.71)		0.96 (0.46-1.96)	
Average dose ≥90%	0.42 (0.19-0.94)		0.53 (0.23-1.21)	
Continuous dose (HR per increment of 10%)	0.86 (0.78-0.94)	.002	0.89 (0.81-0.98)	.01
All-cause death by day 180^b^				
Average dose <50%	1 [Reference]	.04	1 [Reference]	.06
Average dose 50-<90%	0.58 (0.25-1.34)		0.64 (0.27-1.54)	
Average dose ≥90%	0.27 (0.10-0.76)		0.28 (0.10-0.83)	
Continuous dose (HR per increment of 10%)	0.84 (0.74-0.95)	.006	0.84 (0.73-0.95)	.007
**End point (all patients)**				
All-cause death or heart failure readmission by day 180^a^				
Average dose <50%	1 [Reference]	.001	1 [Reference]	.003
Average dose 50-<90%	0.83 (0.61-1.14)		0.81 (0.60-1.11)	
Average dose ≥90%	0.39 (0.23-0.64)		0.41 (0.25-0.68)	
Continuous dose (HR per increment of 10%)	0.89 (0.84-0.94)	<.001	0.90 (0.85-0.95)	<.001
All-cause death by day 180^b^		.05		.05
Average dose <50%	1 [Reference]		1 [Reference]	
Average dose 50-<90%	0.81 (0.52-1.28)		0.86 (0.54-1.35)	
Average dose ≥90%	0.39 (0.18-0.82)		0.39 (0.18-0.84)	
Continuous dose (HR per increment of 10%)	0.90 (0.83-0.97)	.01	0.91 (0.84-0.98)	.02

Abbreviations: HR, hazard ratio; NT-proBNP, N-terminal pro–brain natriuretic peptide.

^a^ Adjusted for baseline diastolic blood pressure, baseline NT-proBNP, ischemic etiology, and edema.

^b^ Adjusted for baseline creatinine, baseline hemoglobin, baseline urea, and baseline NT-proBNP.

Similar results can be seen regarding the association of GDMT dose and mortality (Table 2, Figure 2C and D; eFigure 13, 14, 17, and 18 in Supplement 1, and eTables 2 and 3 in Supplement 1). However, the *P* values for the differences in mortality by dose categories as a time-dependent covariate are just shy of statistical significance (*P* = .06) in the HIC group only (medium-dose group: aHR, 0.64; 95% CI, 0.27-1.54; high-dose group: aHR, 0.28; 95% CI, 0.10-0.83) and *P* = .05 in all patients (medium-dose group: aHR, 0.86; 95% CI, 0.54-1.35; high-dose group: aHR, 0.39; 95% CI, 0.18-0.84). As a continuous time-dependent covariate, each increase of 10% in the average percentage optimal dose was associated with a decrease in 180-day all-cause mortality with an aHR of 0.84 (95% CI, 0.73-0.95; *P* = .007) in the HIC group only and an aHR of 0.91 (95% CI, 0.84-0.98; *P* = .02) in all participants.

### Quality of Life by Average Dose of HF GDMT

Among patients in the HIC group, the EQ-VAS score improved more with increasing dose category at week 2 (eTable 2 in Supplement 1). After covariate adjustment, the change in EQ-VAS from baseline to day 90 was 0.10 (95% CI, −4.88 to 5.07) points higher in the medium-dose category and 3.13 (95% CI, −1.98 to 8.24) points higher in the high-dose category relative to the low-dose category (*P* = .07). Similar outcomes were observed when including all patients and assuming that doses for patients in the UC group were unchanged at week 2 (eTable 3 in Supplement 1). The same tendency was observed when assessing the average percentage optimal dose as a continuous variable (eFigure 19 in Supplement 1), with more improvement in quality of life observed in patients with higher HF GDMT doses.

### Factors Predictive of Successful Implementation of HF Therapies at Week 2

In the HIC group, higher baseline systolic blood pressure, lower New York Heart Association class 1 month before and at randomization, history of diabetes, history of HF, nonischemic HF etiology, lower urea level, a lack of edema, and jugular venous pressure less than 6 cm at prerandomization were associated in a multivariable model with a higher average dose of HF GDMT at week 2 (Table 3). Baseline glucose, sodium, and potassium level had nonlinear associations with the average percentage optimal dose at week 2, with patterns of increasing and then decreasing average dose with increasing concentrations at maxima of approximately 5.4 mmol/L (to convert glucose to milligrams per deciliter, divide by 0.0555), 138 mmol/L (to convert sodium to milliequivalents per liter, divide by 1), and 4 mmol/L (to convert potassium to milliequivalents per liter, divide by 1), respectively.

**Table 3.** Univariable and Multivariable Model Predictors of Average Percentage of Optimal Dose at Week 2 in Patients Assigned High-Intensity Care Only

Predictor	Estimate for unit change of	Univariable results		Multivariable results	
		Est (95% CI)	*P* value	Est (95% CI)	*P* value
Age, y	5	−0.69 (−1.38 to 0.01)	.05	NA	NA
Male sex	Yes vs no	−3.25 (−7.07 to 0.58)	.10		
White race	Yes vs no	−5.36 (−9.33 to −1.40)	.008		
Geographic region	Europe vs not-Europe	−7.52 (−11.71 to −3.33)	<.001		
Baseline systolic BP, mm Hg	5	1.79 (1.09 to 2.48)	<.001	0.92 (0.25 to 1.59)	.007
Baseline pulse, bpm	5	1.15 (0.35 to 1.95)	.005	NA	NA
Baseline eGFR	5	0.72 (0.28 to 1.15)	.001		
NT-proBNP	Doubling	−0.58 (−2.65 to 1.48)	.58		
BMI^a^	2	−0.24 (−0.84 to 0.36)	.44		
LVEF, %	2	0.29 (−0.01 to 0.59)	.06		
NYHA class 1-mo prior	III/IV vs I/II	−2.82 (−5.05 to −0.59)	.01	−2.45 (−4.76 to −0.14)	.04
NYHA class (prerandomization)	III/IV vs I/II	−8.70 (−12.55 to −4.84)	<.001	−4.17 (−8.09 to −0.25)	.04
Angina class II or higher	Yes vs no	−3.07 (−8.63 to 2.50)	.28	NA	NA
History of afib or aflutter present at screening	Yes vs no	−2.42 (−6.21 to 1.38)	.21		
Moderate or severe COPD or asthma	Yes vs no	−13.70 (−27.25 to −0.16)	.05		
History of diabetes	Yes vs no	0.40 (−3.80 to 4.60)	.85	4.73 (0.11 to 9.35)	.05
History of heart failure	Yes vs no	6.08 (0.70 to 11.47)	.03	7.50 (2.18 to 12.81)	.006
Ischemic etiology	Yes vs no	−5.95 (−9.67 to −2.22)	.002	−4.54 (−8.35 to −0.73)	.02
Hemoglobin, g/L^b^	137.00 vs 123.00	−1.37 (−3.26 to 0.53)	.42	NA	NA
	149.00 vs 137.00	−2.23 (−3.92 to −0.54)			
Lymphocytes, %	2	0.55 (0.17 to 0.92)	.005		
White blood cells, 10^9^/L	2	−1.23 (−3.18 to 0.71)	.21		
Total bilirubin, μmol/L	2	−0.48 (−0.80 to −0.16)	.003		
Glucose ≤5.4 mmol/L^c^	2	13.43 (4.99 to 21.88)	.003	15.47 (7.48 to 23.47)	<.001
Glucose >5.4 mmol/L^c^	2	−2.12 (−3.90 to −0.34)		−1.92 (−3.76 to −0.09)	
Creatinine, μmol/L^b^	103.00 vs 87.00	−0.91 (−2.59 to 0.77)	.17	NA	NA
	120.00 vs 103.00	−1.53 (−2.84 to −0.22)		NA	
Sodium, mmol/L^d^	140.00 vs 137.10	0.81 (−0.63 to 2.24)	<.001	0.88 (−0.50 to 2.27)	<.001
	143.00 vs 140.00	−2.11 (−3.78 to −0.44)		−1.67 (−3.26 to −0.08)	
ALT	Doubling	−1.84 (−3.86 to 0.19)	.08	NA	NA
Potassium, mmol/L^d^	4.30 vs 4.00	−1.78 (−3.03 to −0.52)	.01	−0.99 (−2.20 to 0.21)	.01
	4.60 vs 4.30	−3.56 (−5.21 to −1.91)		−2.32 (−3.88 to −0.77)	
Urea, mmol/L	2	−2.50 (−3.53 to −1.46)	<.001	−2.02 (−3.05 to −1.00)	<.001
Total cholesterol, mmol/L	2	7.01 (3.46 to 10.56)	<.001	NA	NA
Edema at prerandomization	2+/3+ vs 0/1+	−10.71 (−18.54 to −2.88)	.008	−8.44 (−16.05 to −0.83)	.03
JVP (prerandomization)	≥6 cm vs <6 cm	−10.66 (−15.73 to −5.59)	<.001	−5.28 (−10.16 to −0.40)	.03

Abbreviations: afib, atrial fibrillation; aflutter, atrial flutter; ALT, alanine aminotransferase; BMI, body mass index; BP, blood pressure; COPD, chronic obstructive pulmonary disease; eGFR, estimated glomerular filtration rate; Est, estimated; LVEF, left ventricular ejection fraction; NA, not applicable; NT-proBNP, N-terminal pro–brain natriuretic peptide; NYHA, New York Heart Association; JVP, jugular venous pressure.

SI conversion factor: To convert hemoglobin to grams per deciliter, divide by 10; to convert white blood cell count to per microliter, divide by 0.001; to convert glucose to milligrams per deciliter, divide by 0.0555; to convert creatinine to milligrams per deciliter, divide by 88.4; to convert potassium and sodium to milliequivalents per liter, divide by 1; to convert urea to milligrams per deciliter, divide by 0.357; to convert ALT to microkatals per liter, multiply by 0.0167; to convert bilirubin to milligrams per deciliter, divide by 17.104; to convert total cholesterol to milligrams per deciliter, divide by 0.0259.

^a^ Calculated as weight in kilograms divided by height in meters squared.

^b^ Nonlinear association modeled as cubic polynomial. Effect sizes for 75th percentile vs median and for median vs 25th percentile are presented.

^c^ Nonlinear association modeled as linear spline.

^d^ Nonlinear association modeled as quadratic polynomial. Effect sizes for 75th percentile vs median and for median vs 25th percentile are presented. Results from linear regression model.

### Occurrence of Adverse Events in the HIC Group

Adverse events occurred less frequently in patients in the HIC group who were prescribed higher GDMT doses at week 2 (eTable 4 in Supplement 1). Among patients in the HIC group, adverse events from week 2 to day 90 were observed in 21 of 39 (53.8%), 98 of 254 (38.6%), and 51 of 222 (23.0%) patients in the low-, medium-, and high-dose categories, respectively (*P* < .001). Of note, cardiac disorders were the most frequent adverse events, observed in 80 patients (15.5%) in the HIC group: 8 patients (20.5%) in the low-dose group, 49 patients (19.3%) in the medium-dose group, and 23 patients (10.4%) in the high-dose group at week 2 (eTable 4 in Supplement 1). Among patients in the HIC group, serious adverse events between week 2 and day 90 occurred in 9 patients (23.1%) in the low-dose group, 37 patients (14.6%) in the medium-dose group, and 21 patients (9.5%) in the high-dose group at week 2 (*P* = .01) (eTable 5 in Supplement 1). The most common serious adverse events were cardiac (5 of 39 [12.8%], 24 of 254 [9.4%], and 12 of 222 [5.4%] in the low-, medium- and high-dose categories, respectively), infections (3 of 39 [7.7%], 6 of 254 [2.4%], and 3 of 222 [1.4%] in the low-, medium- and high-dose categories, respectively) and kidney (2 of 39 [5.1%], 0 of 254 [0%], and 1 of 222 [0.5%] in the low-, medium- and high-dose categories, respectively) without any single serious adverse event occurring significantly more in any group.

### Correlation Between the Doses of Medications

Per protocol, doses of the medications in the 3 classes were ideally to be uptitrated together. The doses of the medications prescribed at week 2 (RASi, BBs and MRAs) were thus moderately correlated (eFigure 20-22 in Supplement 1). The Pearson correlation between BB and RASi doses was 0.43 (*P* < .001), between MRA and RASi doses was 0.32 (*P* < .001), and between BB and MRA doses was 0.23 (*P* < .001).

## Discussion

In this post hoc analysis of the STRONG-HF trial, patients who were not taking optimal GDMT medication doses before discharge from an AHF admission and who were randomly assigned to the HIC arm were uptitrated within 2 weeks of discharge to higher GDMT doses. Approximately one-half these patients were prescribed between 50% to 90%, and 43.1% were prescribed 90% or more, of maximum-recommended GDMT doses.

Clearly, not all patients randomly assigned to the HIC arm were uptitrated to maximal GDMT doses. Our study results suggest that patients with less stable clinical status (lower blood pressure, more congestion, and lower eGFR) before discharge were less likely to get full optimal medications at week 2. The less stable clinical status was also reflected in more adverse events and serious adverse events in those who were not uptitrated to maximal GDMT doses, suggesting that the lack of uptitration may have been associated with the less stable clinical status. This finding is important in informing clinical practice. Physicians treating patients with AHF should recognize that patients who are not currently prescribed optimal GDMT doses before discharge can be rapidly uptitrated to higher doses within 2 weeks after discharge. However, this uptitration may not be complete. Some patients who are more frail, have lower blood pressure, more congestion, and some comorbidities may take longer to uptitrate and in some cases cannot be uptitrated to full GDMT doses.

Given that clinical congestion or an increase in NT-proBNP level were common barriers to uptitration of patients’ GDMT to full recommended doses, one may argue that better decongestion of patients before initiation of high-dose GDMT for HF would have been more efficacious. However, evidence to this effect is not conclusive, and several clinical, biological, and structural phenotypic variables may prevent successful decongestion.^19^ Particularly, patients who had a decrease in eGFR early after an AHF admission had less GDMT uptitration,^20^ and more aggressive predischarge decongestion is known to be associated with more eGFR decreases. In addition, recent randomized clinical trials showed that irrespective of type or intensity of diuretic strategies, there was no association between decongestion and the rate of postdischarge mortality.^20,21^ Finally, there is no evidence from randomized clinical trials to demonstrate that waiting for complete decongestion in order to initiate GDMTs is better than early initiation of GDMTs.^22^ The STRONG-HF trial shows the importance of proper monitoring of congestion through frequent follow-up visits and measurements of NT-proBNP concentration as a tool to decide on which patients will require additional decongestion therapy and hence improve tolerance to GDMT and prevent hospitalizations. This judicious use of decongestion to support rapid uptitration of GDMT seems to be a preferred strategy to enhance decongestion with double and triple diuretics in all patients with AHF.

In the current analysis, higher doses of RASi, BB, and MRA medications were successfully uptitrated under careful safety monitoring, and the ability to uptitrate them was associated with better outcomes. These results pertain to the study’s primary end point, HF readmission or all-cause death at 180 days, with a trend toward improved 180-day survival and significant improvement in quality of life. Because the STRONG-HF trial showed the efficacy and safety of a regimen including both rapid titration of GDMT and close patient follow-up, the current analysis was important in suggesting that the main driver of the benefit of HIC in the STRONG-HF trial was the uptitration of GDMT.

Our study demonstrated that optimization of HF therapies was not associated with an increased risk of safety issues in the patients reaching administration of 90% or more of the HF medications compared with the patients in the lower dose categories. It is, however, difficult to assess whether this lower risk was associated with improvement in HF status or the fact that patients who succeeded in getting optimal medications were in a better clinical situation.

### Limitations

The present study has a few limitations. First, benefits of combined HF therapies have been tested, and analyses could not discriminate benefits of each class of HF medication. Second, patients were not randomly assigned to the dose groups examined. There may be important differences in characteristics and event risk among patients who were and were not uptitrated at 2 weeks. Although results were adjusted for patient status at baseline, no randomized, dose-achieved comparisons studies like the present study can reveal associations, but the findings may be confounded by factors relating to inability to uptitrate medications. Lastly, our study could not assess the effects of 2 novel medications: angiotensin receptor-neprilysin inhibitors and sodium-glucose transport protein 2 inhibitors (SGLT2is). RASi medications were prescribed as per each center’s preference, and SGLT2i medications were not prescribed during most of the study due to lack of approval.

## Conclusions

In summary, this post hoc secondary analysis from the STRONG-HF study demonstrated that higher achieved doses of HF GDMT medications were associated with better outcomes and greater improvement of quality of life, with the best results seen in patients treated with an average dose of 90% or more of maximally recommended doses. Therefore, when patients can tolerate higher doses of GDMT, all efforts should be made to rapidly uptitrate patients with AHF to optimal doses of the 3 and (likely) 4 pillars of HF medications, including RASis, BBs, MRAs and SGLT2is.

## Notes

Supplement 1. **eTable 1. **Changes From Week 2 to Day 90 in Vital Signs and Laboratory Data by Average Optimal Dose Categories at Week 2 **eTable 2. **Clinical Outcomes by Average Percentage Optimal Dose Categories at Week 2 in High-Intensity Care Patients **eTable 3.** Clinical Outcomes by Average Percentage Optimal Dose Categories at Week 2 in All Subjects **eTable 4.** Treatment Emergent Adverse Events by Average Percentage Optimal Dose Categories at Week 2 **eTable 5.** Treatment Emergent Serious Adverse Events by Average Percentage Optimal Dose Categories at Week 2 **eFigure 1.** Change in NT-ProBNP by Average Percentage Optimal Dose Group at Week 2—High-Intensity Care Subjects **eFigure 2.** Change in NT-ProBNP by Average Percentage Optimal Dose Group at Week 2 (or Post-Rand For Usual Care)- All Subjects **eFigure 3.** Change in Potassium by Average Percentage Optimal Dose Group at Week 2—High-Intensity Care Subjects **eFigure 4.** Change in Potassium by Average Percentage Optimal Dose Group at Week 2 (or Post-Rand for Usual Care)—All Subjects **eFigure 5.** Change in Creatinine by Average Percentage Optimal Dose Group at Week 2—High-Intensity Care Subjects **eFigure 6.** Change in Creatinine by Average Percentage Optimal Dose Group at Week 2 (or Post-Rand for Usual Care)—All Subjects **eFigure 7.** Change in Hemoglobin by Average Percentage Optimal Dose Group at Week 2—High-Intensity Care Subjects **eFigure 8.** Change in Hemoglobin by Average Percentage Optimal Dose Group at Week 2 (or Post-Rand for Usual Care)—All Subjects **eFigure 9.** Change in eGFR by average percentage optimal dose group at Week 2—High-Intensity Care Subjects **eFigure 10.** Change in eGFR by Average Percentage Optimal Dose Group at Week 2 (or Post-Rand for Usual Care)—All Subjects **eFigure 11.** Kaplan-Meier Curves for All-Cause Mortality or Heart Failure Readmission Through Day 180 by Average Percentage Optimal Dose Category at Week 2—High-Intensity Care Subjects **eFigure 12.** Kaplan-Meier Curves for All-Cause Mortality or Heart Failure Readmission Through Day 180 by Average Percentage Optimal Dose Category at Week 2—All Subjects **eFigure 13.** Kaplan-Meier Curves for All-Cause Mortality Through Day 180 by Average Optimal Percentage Dose Category at Week 2—High-Intensity Care Subjects **eFigure 14.** Kaplan-Meier Curves for All-Cause Mortality Through Day 180 by Average Percentage Optimal Dose Category at Week 2 (or Post-Rand for Usual Care)—All Subjects **eFigure 15.** Relative Hazard of All-Cause Mortality or Heart Failure Readmission Through Day 180 by Average Percentage Optimal Dose at Week 2 in HIC Patients **eFigure 16.** Relative Hazard of All-Cause Mortality or Heart Failure Readmission Through Day 180 by Average Percentage Optimal Dose at Week 2 in All Subjects **eFigure 17.** Relative Hazard of All-Cause Mortality Through Day 180 by Average Percentage Optimal Dose at Week 2 in HIC Patients **eFigure 18.** Relative Hazard of All-Cause Mortality Through Day 180 by Average Percentage Optimal Dose at Week 2 in All Patients **eFigure 19.** Change From Baseline in EQ-VAS to Day 90 by Average Percentage Optimal Dose at Week 2 in HIC Patients **eFigure 20.** Bubble Plot of Percent Optimal Dose of RASi by Beta-Blockers at Week 2 **eFigure 21.** Bubble Plot of Percent Optimal Dose of RASi by MRA at Week 2 **eFigure 22.** Bubble Plot of Percent Optimal Dose of β-Blockers by MRA at Week 2

Supplement 2.
Data Sharing Statement.
