# Supplementary material for: Optimization of Evidence-Based Heart Failure Medications After an Acute Heart Failure Admission: A Secondary Analysis of the STRONG-HF Randomized Clinical Trial
Source: JAMA Cardiol. 2023 Dec 27;9(2):114–24. doi: 10.1001/jamacardio.2023.4553 (PMC10753435; doi:10.1001/jamacardio.2023.4553)
Supplement: Supplement 1. — eTable 1. Changes From Week 2 to Day 90 in Vital Signs and Laboratory Data by Average Optimal Dose Categories at Week 2 eTable 2. Clinical Outcomes by Average Percentage Optimal Dose Categories at Week 2 in High-Intensity Care Patients eTable 3. Clinical Outcomes by Average Percentage Optimal Dose Categories at Week 2 in All Subjects eTable 4. Treatment Emergent Adverse Events by Average Percentage Optimal Dose Categories at Week 2 eTable 5. Treatment Emergent Serious Adverse Events by Average Percentage Optimal Dose Categories at Week 2 eFigure 1. Change in NT-ProBNP by Average Percentage Optimal Dose Group at Week 2—High-Intensity Care Subjects eFigure 2. Change in NT-ProBNP by Average Percentage Optimal Dose Group at Week 2 (or Post-Rand For Usual Care)- All Subjects eFigure 3. Change in Potassium by Average Percentage Optimal Dose Group at Week 2—High-Intensity Care Subjects eFigure 4. Change in Potassium by Average Percentage Optimal Dose Group at Week 2 (or Post-Rand for Usual Care)—All Subjects eFigure 5. Change in Creatinine by Average Percentage Optimal Dose Group at Week 2—High-Intensity Care Subjects eFigure 6. Change in Creatinine by Average Percentage Optimal Dose Group at Week 2 (or Post-Rand for Usual Care)—All Subjects eFigure 7. Change in Hemoglobin by Average Percentage Optimal Dose Group at Week 2—High-Intensity Care Subjects eFigure 8. Change in Hemoglobin by Average Percentage Optimal Dose Group at Week 2 (or Post-Rand for Usual Care)—All Subjects eFigure 9. Change in eGFR by average percentage optimal dose group at Week 2—High-Intensity Care Subjects eFigure 10. Change in eGFR by Average Percentage Optimal Dose Group at Week 2 (or Post-Rand for Usual Care)—All Subjects eFigure 11. Kaplan-Meier Curves for All-Cause Mortality or Heart Failure Readmission Through Day 180 by Average Percentage Optimal Dose Category at Week 2—High-Intensity Care Subjects eFigure 12. Kaplan-Meier Curves for All-Cause Mortality or Heart Failure Readmission Through Day [file jamacardiol-e234553-s001.pdf]

Supplementary Online Content

Cotter G, Deniau B, Davison B, et al. Optimization of evidence-based heart failure medications after an acute heart failure admission: a secondary analysis of the STRONG-HF randomized clinical trial. *JAMA Cardiol*. Published online December 27, 2023. doi:10.1001/jamacardio.2023.4553

eTable 1. Changes From Week 2 to Day 90 in Vital Signs and Laboratory Data by Average Optimal Dose Categories at Week 2

eTable 2. Clinical Outcomes by Average Percentage Optimal Dose Categories at Week 2 in High-Intensity Care Patients

eTable 3. Clinical Outcomes by Average Percentage Optimal Dose Categories at Week 2 in All Subjects

eTable 4. Treatment Emergent Adverse Events by Average Percentage Optimal Dose Categories at Week 2

eTable 5. Treatment Emergent Serious Adverse Events by Average Percentage Optimal Dose Categories at Week 2

eFigure 1. Change in NT-ProBNP by Average Percentage Optimal Dose Group at Week 2—High-Intensity Care Subjects

eFigure 2. Change in NT-ProBNP by Average Percentage Optimal Dose Group at Week 2 (or Post-Rand For Usual Care)- All Subjects

eFigure 3. Change in Potassium by Average Percentage Optimal Dose Group at Week 2—High-Intensity Care Subjects

eFigure 4. Change in Potassium by Average Percentage Optimal Dose Group at Week 2 (or Post-Rand for Usual Care)—All Subjects

eFigure 5. Change in Creatinine by Average Percentage Optimal Dose Group at Week 2—High-Intensity Care Subjects

eFigure 6. Change in Creatinine by Average Percentage Optimal Dose Group at Week 2 (or Post-Rand for Usual Care)—All Subjects

eFigure 7. Change in Hemoglobin by Average Percentage Optimal Dose Group at Week 2—High-Intensity Care Subjects

eFigure 8. Change in Hemoglobin by Average Percentage Optimal Dose Group at Week 2 (or Post-Rand for Usual Care)—All Subjects

eFigure 9. Change in eGFR by average percentage optimal dose group at Week 2—High-Intensity Care Subjects

© 2023 Cotter G et al. *JAMA Cardiology.*

eFigure 10. Change in eGFR by Average Percentage Optimal Dose Group at Week 2 (or Post-Rand for Usual Care)—All Subjects

eFigure 11. Kaplan-Meier Curves for All-Cause Mortality or Heart Failure Readmission Through Day 180 by Average Percentage Optimal Dose Category at Week 2—High-Intensity Care Subjects

eFigure 12. Kaplan-Meier Curves for All-Cause Mortality or Heart Failure Readmission Through Day 180 by Average Percentage Optimal Dose Category at Week 2—All Subjects

eFigure 13. Kaplan-Meier Curves for All-Cause Mortality Through Day 180 by Average Optimal Percentage Dose Category at Week 2—High-Intensity Care Subjects

eFigure 14. Kaplan-Meier Curves for All-Cause Mortality Through Day 180 by Average Percentage Optimal Dose Category at Week 2 (or Post-Rand for Usual Care)—All Subjects

eFigure 15. Relative Hazard of All-Cause Mortality or Heart Failure Readmission Through Day 180 by Average Percentage Optimal Dose at Week 2 in HIC Patients

eFigure 16. Relative Hazard of All-Cause Mortality or Heart Failure Readmission Through Day 180 by Average Percentage Optimal Dose at Week 2 in All Subjects

eFigure 17. Relative Hazard of All-Cause Mortality Through Day 180 by Average Percentage Optimal Dose at Week 2 in HIC Patients

eFigure 18. Relative Hazard of All-Cause Mortality Through Day 180 by Average Percentage Optimal Dose at Week 2 in All Patients

eFigure 19. Change From Baseline in EQ-VAS to Day 90 by Average Percentage Optimal Dose at Week 2 in HIC Patients

eFigure 20. Bubble Plot of Percent Optimal Dose of RASi by Beta-Blockers at Week 2

eFigure 21. Bubble Plot of Percent Optimal Dose of RASi by MRA at Week 2

eFigure 22. Bubble Plot of Percent Optimal Dose of β-Blockers by MRA at Week 2

© 2023 Cotter G et al. *JAMA Cardiology.*

This supplementary material has been provided by the authors to give readers additional information about their work.

© 2023 Cotter G et al. *JAMA Cardiology.*

eTable 1. Changes from week 2 to day 90 in vital signs and laboratory data by average optimal dose categories at week 2

Parameter	Statistic	Average Dose <50% (n= 39)	Average Dose >=50-<90% (n=254)	Average Dose >=90% (n=222)
Vital Signs				
Systolic Blood Pressure, mmHg				
Week 2	Mean (SD)	110.60 (15.62)	119.81 (16.28)	124.90 (13.64)
Day 90	Mean (SD)	121.62 (17.52)	118.94 (14.98)	118.14 (16.28)
Adjusted Mean Change	LS Mean (SE)	4.38 (2.40)	-1.65 (0.91)	-4.93 (0.96)
LS Mean Difference	LS Mean Diff (95% CI)	[ref]	-6.03 (-11.05, -1.00)	-9.31 (-14.43, -4.18)
P-value (vs. reference)		--	0.02	<0.001
Diastolic Blood Pressure, mmHg				
Week 2	Mean (SD)	68.34 (11.53)	74.28 (10.77)	78.13 (9.90)
Day 90	Mean (SD)	73.65 (9.58)	73.42 (10.38)	74.53 (10.35)
Adjusted Mean Change	LS Mean (SE)	1.18 (1.63)	-1.44 (0.62)	-1.99 (0.65)
LS Mean Difference	LS Mean Diff (95% CI)	[ref]	-2.62 (-6.03, 0.78)	-3.18 (-6.66, 0.31)
P-value (vs. reference)		--	0.13	0.07
Heart Rate, beats/min				
Week 2	Mean (SD)	74.31 (14.74)	74.00 (13.17)	76.26 (10.11)
Day 90	Mean (SD)	71.32 (14.21)	71.11 (13.55)	71.30 (12.79)
Adjusted Mean Change	LS Mean (SE)	-3.24 (2.09)	-2.89 (0.81)	-3.98 (0.85)
LS Mean Difference	LS Mean Diff (95% CI)	[ref]	0.36 (-4.05, 4.77)	-0.74 (-5.18, 3.70)
P-value (vs. reference)		--	0.87	0.74
Respiratory Rate, breaths/min				
Week 2	Mean (SD)	18.18 (2.26)	17.62 (2.23)	16.83 (2.33)
Day 90	Mean (SD)	18.03 (2.80)	17.62 (2.45)	16.57 (2.15)
Adjusted Mean Change	LS Mean (SE)	0.26 (0.32)	0.13 (0.12)	-0.38 (0.13)
LS Mean Difference	LS Mean Diff (95% CI)	[ref]	-0.13 (-0.79, 0.53)	-0.64 (-1.31, 0.03)

© 2023 Cotter G et al. *JAMA Cardiology.*

Parameter	Statistic	Average Dose <50% (n= 39)	Average Dose >=50-<90% (n=254)	Average Dose >=90% (n=222)
P-value (vs. reference)		--	0.70	0.06
Weight, kg				
Week 2	Mean (SD)	80.12 (20.21)	82.81 (19.42)	77.67 (20.99)
Day 90	Mean (SD)	77.72 (19.10)	82.59 (19.42)	77.11 (20.63)
Adjusted Mean Change	LS Mean (SE)	-2.85 (0.65)	-0.34 (0.25)	-0.98 (0.26)
LS Mean Difference	LS Mean Diff (95% CI)	[ref]	2.51 (1.14, 3.87)	1.87 (0.49, 3.24)
P-value (vs. reference)		--	<0.001	0.008
Local Laboratory				
Hemoglobin, g/L				
Week 2	Mean (SD)	129.1 (19.83)	137.4 (19.26)	133.4 (16.95)
Day 90	Mean (SD)	127.3 (17.69)	134.0 (17.98)	130.6 (15.54)
Adjusted Mean Change	LS Mean (SE)	-3.88 (2.20)	-3.41 (0.83)	-3.22 (0.86)
LS Mean Difference	LS Mean Diff (95% CI)	[ref]	0.47 (-4.16, 5.10)	0.66 (-3.97, 5.29)
P-value (vs. reference)		--	0.84	0.78
White Blood Cells,10/L				
Week 2	Mean (SD)	6.74 (2.18)	7.24 (1.94)	6.70 (1.82)
Day 90	Mean (SD)	6.96 (1.76)	7.12 (1.93)	6.57 (1.61)
Adjusted Mean Change	LS Mean (SE)	-0.07 (0.26)	-0.01 (0.10)	-0.22 (0.10)
LS Mean Difference	LS Mean Diff (95% CI)	[ref]	0.07 (-0.48, 0.62)	-0.15 (-0.70, 0.40)
P-value (vs. reference)		--	0.81	0.60
Lymphocytes,%				
Week 2	Mean (SD)	25.88 (8.06)	27.67 (8.83)	28.66 (8.88)
Day 90	Mean (SD)	25.17 (9.01)	27.73 (8.82)	29.02 (8.38)
Adjusted Mean Change	LS Mean (SE)	-1.29 (1.32)	-0.08 (0.50)	0.36 (0.53)
LS Mean Difference	LS Mean Diff (95% CI)	[ref]	1.21 (-1.57, 3.98)	1.64 (-1.16, 4.45)
P-value (vs. reference)		--	0.39	0.25
Glucose, mmol/L				

© 2023 Cotter G et al. *JAMA Cardiology.*

Parameter	Statistic	Average Dose <50% (n= 39)	Average Dose >=50-<90% (n=254)	Average Dose >=90% (n=222)
Week 2	Mean (SD)	7.03 (3.63)	6.50 (2.58)	5.79 (1.95)
Day 90	Mean (SD)	6.34 (2.25)	6.74 (2.85)	5.85 (1.90)
Adjusted Mean Change	LS Mean (SE)	-0.19 (0.36)	0.34 (0.13)	-0.12 (0.14)
LS Mean Difference	LS Mean Diff (95% CI)	[ref]	0.53 (-0.21, 1.28)	0.07 (-0.68, 0.81)
P-value (vs. reference)		--	0.16	0.86
Sodium, mmol/L				
Week 2	Mean (SD)	140.1 (5.18)	140.7 (4.23)	139.6 (3.69)
Day 90	Mean (SD)	141.3 (5.34)	141.1 (4.71)	139.7 (3.37)
Adjusted Mean Change	LS Mean (SE)	1.09 (0.61)	0.58 (0.23)	-0.10 (0.25)
LS Mean Difference	LS Mean Diff (95% CI)	[ref]	-0.51 (-1.80, 0.78)	-1.19 (-2.49, 0.10)
P-value (vs. reference)		--	0.44	0.07
Potassium, mmol/L				
Week 2	Mean (SD)	4.75 (0.66)	4.60 (0.51)	4.48 (0.38)
Day 90	Mean (SD)	4.65 (0.47)	4.55 (0.54)	4.65 (0.37)
Adjusted Mean Change	LS Mean (SE)	0.02 (0.08)	-0.03 (0.03)	0.12 (0.03)
LS Mean Difference	LS Mean Diff (95% CI)	[ref]	-0.05 (-0.21, 0.11)	0.10 (-0.06, 0.27)
P-value (vs. reference)		--	0.53	0.22
Urea, mmol/L				
Week 2	Mean (SD)	10.63 (5.85)	8.32 (3.89)	6.80 (2.62)
Day 90	Mean (SD)	10.00 (5.66)	8.35 (3.99)	6.86 (2.90)
Adjusted Mean Change	LS Mean (SE)	0.35 (0.52)	0.11 (0.19)	-0.58 (0.20)
LS Mean Difference	LS Mean Diff (95% CI)	[ref]	-0.24 (-1.31, 0.84)	-0.93 (-2.03, 0.18)
P-value (vs. reference)		--	0.67	0.10
eGFR, ml/min/1.732				
Week 2	Mean (SD)	56.35 (24.49)	62.30 (22.44)	68.33 (21.28)
Day 90	Mean (SD)	59.95 (22.56)	62.86 (22.64)	66.79 (21.45)
Adjusted Mean Change	LS Mean (SE)	2.91 (2.45)	0.23 (0.92)	-0.37 (0.96)
LS Mean Difference	LS Mean Diff (95% CI)	[ref]	-2.69 (-7.82, 2.45)	-3.28 (-8.48, 1.92)

© 2023 Cotter G et al. *JAMA Cardiology.*

Parameter	Statistic	Average Dose <50% (n= 39)	Average Dose >=50-<90% (n=254)	Average Dose >=90% (n=222)
P-value (vs. reference)		--	0.30	0.22
Uric Acid, umol/L				
Week 2	Mean (SD)	433.0 (105.9)	412.8 (123.0)	405.4 (83.97)
Day 90	Mean (SD)	412.7 (115.0)	392.2 (110.0)	404.4 (94.76)
Adjusted Mean Change	LS Mean (SE)	-12.2 (17.23)	-17.8 (6.55)	-5.83 (6.48)
LS Mean Difference	LS Mean Diff (95% CI)	[ref]	-5.60 (-41.87, 30.66)	6.33 (-29.88, 42.54)
P-value (vs. reference)		--	0.76	0.73
AST, U/L				
Week 2	Mean (SD)	25.73 (13.04)	26.69 (13.30)	23.24 (16.42)
Day 90	Mean (SD)	33.17 (46.71)	24.48 (10.91)	24.27 (44.05)
Adjusted Mean Change	LS Mean (SE)	7.88 (5.32)	-2.15 (2.09)	1.36 (2.19)
LS Mean Difference	LS Mean Diff (95% CI)	[ref]	-10.04 (-21.27, 1.20)	-6.53 (-17.85, 4.79)
P-value (vs. reference)		--	0.08	0.26
ALT, U/L				
Week 2	Mean (SD)	22.90 (12.75)	28.09 (30.68)	22.42 (21.50)
Day 90	Mean (SD)	41.55 (115.6)	23.44 (12.30)	20.92 (13.71)
Adjusted Mean Change	LS Mean (SE)	19.50 (6.24)	-1.99 (2.47)	-3.70 (2.58)
LS Mean Difference	LS Mean Diff (95% CI)	[ref]	-21.49 (-34.69, -8.29)	-23.21 (-36.46, -9.95)
P-value (vs. reference)		--	0.002	<0.001
Total Bilirubin, umol/L				
Week 2	Mean (SD)	15.94 (8.43)	15.70 (9.77)	12.31 (5.44)
Day 90	Mean (SD)	15.65 (9.54)	15.24 (9.96)	12.91 (7.33)
Adjusted Mean Change	LS Mean (SE)	0.13 (1.58)	0.68 (0.59)	-0.44 (0.62)
LS Mean Difference	LS Mean Diff (95% CI)	[ref]	0.55 (-2.76, 3.86)	-0.56 (-3.92, 2.79)
P-value (vs. reference)		--	0.74	0.74

© 2023 Cotter G et al. *JAMA Cardiology.*

Parameter	Statistic	Average Dose <50% (n= 39)	Average Dose >=50-<90% (n=254)	Average Dose >=90% (n=222)
Total Cholesterol, mmol/L				
Week 2	Mean (SD)	4.13 (1.18)	4.23 (1.07)	4.59 (1.09)
Day 90	Mean (SD)	4.35 (1.23)	4.38 (1.13)	4.46 (0.90)
Adjusted Mean Change	LS Mean (SE)	0.15 (0.16)	0.05 (0.06)	-0.05 (0.06)
LS Mean Difference	LS Mean Diff (95% CI)	[ref]	-0.09 (-0.43, 0.25)	-0.20 (-0.54, 0.14)
P-value (vs. reference)		--	0.59	0.25
NT-proBNP, pg/mL				
Week 2	Geo. Mean (95% CI)	3458.3 (2467.4, 4847.0)	2885.9 (2551.8, 3263.9)	1661.1 (1459.0, 1891.3)
Day 90	Geo. Mean (95% CI)	2325.1 (1657.0, 3262.6)	1635.3 (1409.7, 1897.0)	978.0 (840.2, 1138.3)
Adjusted Mean Change[a]	LS Mean (SE)	0.73 (0.18)	0.64 (0.07)	0.51 (0.07)
LS Mean Difference	LS Mean Diff (95% CI)	[ref]	0.87 (0.60, 1.27)	0.70 (0.48, 1.03)
P-value (vs. reference)		--	0.48	0.07

[a] Statistics are estimated based on an Analysis of Covariance (ANCOVA) model adjusted for Week 2 value.

© 2023 Cotter G et al. *JAMA Cardiology.*

eTable 2. Clinical outcomes by average percentage optimal dose categories at week 2 in high intensity care patients

		Unadjusted		Adjusted	
Endpoint	n/N (KM%)	HR (95% CI)	P-value	HR (95% CI)	P-value
All-cause death or heart failure readmission by day 180			0.47		0.75
Average Dose <50%	6/31 (18.9%)	[ref]		[ref]	
Average Dose >=50% - <90%	31/234 (13.3%)	0.70 (0.27, 1.84)		0.76 (0.29, 2.01)	
Average Dose >=90%	23/214 (11.3%)	0.55 (0.20, 1.49)		0.68 (0.25, 1.86)	
Test of Proportional Hazards			0.12		
All-cause death by day 180			0.81		0.96
Average Dose <50%	3/34 (9.4%)	[ref]		[ref]	
Average Dose >=50% - <90%	14/235 (6.6%)	0.70 (0.18, 2.73)		0.94 (0.22, 3.99)	
Average Dose >=90%	12/214 (6.1%)	0.63 (0.16, 2.54)		0.80 (0.18, 3.58)	
Test of Proportional Hazards			0.87		
	LS-Mean (SE)	LS-Mean Difference (95% CI)		LS-Mean Difference (95% CI)	
EQ-VAS Change from Baseline to Visit 7			0.001		0.07
Average Dose <50%	7.01 (2.42)	[ref]		[ref]	
Average Dose >=50% - <90%	8.57 (0.94)	1.56 (-3.50, 6.61)		0.10 (-4.88, 5.07)	
Average Dose >=90%	13.51 (1.01)	6.50 (1.39, 11.61)		3.13 (-1.98, 8.24)	

© 2023 Cotter G et al. *JAMA Cardiology.*

eTable 3. Clinical outcomes by average percentage optimal dose categories at week 2 in all subjects

		Unadjusted		Adjusted	
Endpoint	n/N (KM%)	HR (95% CI)	P-value	HR (95% CI)	P-value
All-cause death or heart failure readmission by day 180			0.009		0.02
Average Dose <50%	92/444 (22.0%)	[ref]		[ref]	
Average Dose >=50% - <90%	49/317 (16.6%)	0.73 (0.50, 1.07)		0.71 (0.49, 1.04)	
Average Dose >=90%	23/215 (11.3%)	0.46 (0.27, 0.77)		0.50 (0.29, 0.84)	
Test of Proportional Hazards			0.17		
All-cause death by day 180			0.23		0.36
Average Dose <50%	42/446 (9.8%)	[ref]		[ref]	
Average Dose >=50% - <90%	20/318 (7.2%)	0.70 (0.39, 1.24)		0.76 (0.42, 1.35)	
Average Dose >=90%	12/215 (6.1%)	0.58 (0.28, 1.19)		0.62 (0.30, 1.29)	
Test of Proportional Hazards			0.04		
	LS-Mean (SE)	LS-Mean Difference (95% CI)		LS-Mean Difference (95% CI)	
EQ-VAS Change from Baseline to Visit 7			<0.001		<0.001
Average Dose <50%	6.89 (0.98)	[ref]		[ref]	
Average Dose >=50% - <90%	8.68 (0.96)	1.80 (-0.26, 3.85)		1.62 (-0.38, 3.62)	
Average Dose >=90%	12.40 (1.14)	5.51 (3.20, 7.82)		5.21 (2.97, 7.46)	

© 2023 Cotter G et al. *JAMA Cardiology.*

eTable 4. Treatment emergent adverse events by average percentage optimal dose categories at week 2

System Organ Class<br>Preferred Term	Statistic	Average Dose <50% (N=39)	Average Dose >=50-<90% (N=254)	Average Dose >=90% (N=222)	Trend P-value[#]
Any Adverse Event	n (%)	21 (53.8%)	98 (38.6%)	51 (23.0%)	<0.001
Blood And Lymphatic System Disorders	n (%)	1 (2.6%)	0	2 (0.9%)	
Anaemia	n (%)	1 (2.6%)	0	0	
Anaemia Of Chronic Disease	n (%)	0	0	1 (0.5%)	
Febrile Neutropenia	n (%)	0	0	1 (0.5%)	
Leukocytosis	n (%)	0	0	1 (0.5%)	
Cardiac Disorders	n (%)	8 (20.5%)	49 (19.3%)	23 (10.4%)	0.008
Acute Coronary Syndrome	n (%)	0	1 (0.4%)	0	
Acute Myocardial Infarction	n (%)	1 (2.6%)	1 (0.4%)	1 (0.5%)	
Angina Unstable	n (%)	0	1 (0.4%)	0	
Atrial Fibrillation	n (%)	0	2 (0.8%)	0	
Atrioventricular Block Complete	n (%)	1 (2.6%)	0	0	
Bradycardia	n (%)	0	0	2 (0.9%)	
Cardiac Failure	n (%)	5 (12.8%)	43 (16.9%)	19 (8.6%)	
Cardiac Failure Chronic	n (%)	0	2 (0.8%)	1 (0.5%)	
Myocardial Infarction	n (%)	0	0	1 (0.5%)	
Ventricular Tachycardia	n (%)	1 (2.6%)	0	0	
Gastrointestinal Disorders	n (%)	0	6 (2.4%)	4 (1.8%)	
Abdominal Pain	n (%)	0	1 (0.4%)	0	
Abdominal Pain Upper	n (%)	0	0	1 (0.5%)	
Constipation	n (%)	0	0	1 (0.5%)	
Diarrhoea	n (%)	0	2 (0.8%)	2 (0.9%)	
Dry Mouth	n (%)	0	0	1 (0.5%)	
Dyspepsia	n (%)	0	1 (0.4%)	1 (0.5%)	
Eructation	n (%)	0	1 (0.4%)	0	

© 2023 Cotter G et al. *JAMA Cardiology.*

System Organ Class<br>Preferred Term	Statistic	Average Dose <50% (N=39)	Average Dose >=50-<90% (N=254)	Average Dose >=90% (N=222)	Trend P-value[#]
Gastrointestinal Motility Disorder	n (%)	0	1 (0.4%)	0	
Haemorrhoids	n (%)	0	1 (0.4%)	0	
General Disorders And Administration Site Conditions	n (%)	1 (2.6%)	4 (1.6%)	2 (0.9%)	
Asthenia	n (%)	0	1 (0.4%)	1 (0.5%)	
Fatigue	n (%)	0	1 (0.4%)	0	
Sudden Death	n (%)	1 (2.6%)	2 (0.8%)	1 (0.5%)	
Hepatobiliary Disorders	n (%)	0	0	1 (0.5%)	
Hepatitis Acute	n (%)	0	0	1 (0.5%)	
Infections And Infestations	n (%)	3 (7.7%)	16 (6.3%)	4 (1.8%)	
Bronchitis	n (%)	0	1 (0.4%)	0	
Bursitis Infective	n (%)	0	1 (0.4%)	0	
Corona Virus Infection	n (%)	0	1 (0.4%)	3 (1.4%)	
Gastroenteritis	n (%)	0	1 (0.4%)	0	
Influenza	n (%)	0	0	0	
Lower Respiratory Tract Infection	n (%)	1 (2.6%)	0	0	
Nasopharyngitis	n (%)	0	5 (2.0%)	0	
Pneumonia	n (%)	2 (5.1%)	2 (0.8%)	0	
Pneumonia Viral	n (%)	0	0	1 (0.5%)	
Pyelonephritis	n (%)	0	0	1 (0.5%)	
Respiratory Tract Infection Viral	n (%)	0	3 (1.2%)	0	
Urinary Tract Infection	n (%)	0	1 (0.4%)	0	
Viral Infection	n (%)	0	1 (0.4%)	0	
Vulvovaginal Mycotic Infection	n (%)	0	1 (0.4%)	0	
Injury, Poisoning And Procedural Complications	n (%)	0	4 (1.6%)	1 (0.5%)	
Contusion	n (%)	0	1 (0.4%)	1 (0.5%)	
Fall	n (%)	0	1 (0.4%)	0	
Humerus Fracture	n (%)	0	1 (0.4%)	0	
Toxicity To Various Agents	n (%)	0	1 (0.4%)	0	

© 2023 Cotter G et al. *JAMA Cardiology.*

System Organ Class<br>Preferred Term	Statistic	Average Dose <50% (N=39)	Average Dose >=50-<90% (N=254)	Average Dose >=90% (N=222)	Trend P-value[#]
Investigations	n (%)	2 (5.1%)	5 (2.0%)	5 (2.3%)	0.64
Blood Creatinine Increased	n (%)	1 (2.6%)	0	1 (0.5%)	
Blood Potassium Increased	n (%)	1 (2.6%)	1 (0.4%)	0	
Blood Pressure Increased	n (%)	0	3 (1.2%)	1 (0.5%)	
Glomerular Filtration Rate Decreased	n (%)	0	0	2 (0.9%)	
N-Terminal Prohormone Brain Natriuretic Peptide Increased	n (%)	0	1 (0.4%)	1 (0.5%)	
Platelet Count Decreased	n (%)	1 (2.6%)	0	0	
Metabolism And Nutrition Disorders	n (%)	5 (12.8%)	9 (3.5%)	3 (1.4%)	0.002
Decreased Appetite	n (%)	0	1 (0.4%)	0	
Dehydration	n (%)	0	1 (0.4%)	0	
Hyperkalaemia	n (%)	4 (10.3%)	6 (2.4%)	3 (1.4%)	0.01
Hyperuricaemia	n (%)	1 (2.6%)	0	0	
Hypoglycaemia	n (%)	1 (2.6%)	0	0	
Hypokalaemia	n (%)	0	1 (0.4%)	0	
Type 2 Diabetes Mellitus	n (%)	0	1 (0.4%)	0	
Musculoskeletal And Connective Tissue Disorders	n (%)	0	3 (1.2%)	3 (1.4%)	
Arthritis	n (%)	0	1 (0.4%)	1 (0.5%)	
Back Pain	n (%)	0	0	1 (0.5%)	
Gouty Arthritis	n (%)	0	1 (0.4%)	0	
Muscular Weakness	n (%)	0	1 (0.4%)	1 (0.5%)	
Osteochondrosis	n (%)	0	0	1 (0.5%)	
Nervous System Disorders	n (%)	0	7 (2.8%)	5 (2.3%)	0.82
Cerebrovascular Accident	n (%)	0	2 (0.8%)	1 (0.5%)	1.00
Dizziness	n (%)	0	3 (1.2%)	0	
Dizziness Exertional	n (%)	0	1 (0.4%)	0	
Dizziness Postural	n (%)	0	1 (0.4%)	0	
Dysgeusia	n (%)	0	0	1 (0.5%)	
Headache	n (%)	0	0	2 (0.9%)	

© 2023 Cotter G et al. *JAMA Cardiology.*

System Organ Class<br>Preferred Term	Statistic	Average Dose <50% (N=39)	Average Dose >=50-<90% (N=254)	Average Dose >=90% (N=222)	Trend P-value[#]
Hemianopia Homonymous	n (%)	0	1 (0.4%)	0	
Loss Of Consciousness	n (%)	0	1 (0.4%)	0	
Paraesthesia	n (%)	0	0	2 (0.9%)	
Somnolence	n (%)	0	1 (0.4%)	0	
Renal And Urinary Disorders	n (%)	4 (10.3%)	6 (2.4%)	4 (1.8%)	0.05
Acute Kidney Injury	n (%)	1 (2.6%)	1 (0.4%)	0	
Chronic Kidney Disease	n (%)	1 (2.6%)	0	0	
Renal Failure	n (%)	0	0	1 (0.5%)	
Renal Impairment	n (%)	2 (5.1%)	5 (2.0%)	3 (1.4%)	
Reproductive System And Breast Disorders	n (%)	0	0	1 (0.5%)	
Gynaecomastia	n (%)	0	0	1 (0.5%)	
Respiratory, Thoracic And Mediastinal Disorders	n (%)	1 (2.6%)	2 (0.8%)	1 (0.5%)	
Cough	n (%)	0	1 (0.4%)	0	
Dyspnoea	n (%)	1 (2.6%)	0	0	
Pulmonary Embolism	n (%)	0	0	1 (0.5%)	
Pulmonary Oedema	n (%)	0	1 (0.4%)	0	
Skin And Subcutaneous Tissue Disorders	n (%)	0	1 (0.4%)	1 (0.5%)	
Rash	n (%)	0	0	1 (0.5%)	
Stasis Dermatitis	n (%)	0	1 (0.4%)	0	
Surgical And Medical Procedures	n (%)	0	1 (0.4%)	1 (0.5%)	
Chemotherapy	n (%)	0	0	1 (0.5%)	
High Frequency Ablation	n (%)	0	1 (0.4%)	0	
Vascular Disorders	n (%)	2 (5.1%)	14 (5.5%)	4 (1.8%)	0.07
Hypertension	n (%)	0	0	1 (0.5%)	
Hypertensive Crisis	n (%)	0	3 (1.2%)	1 (0.5%)	
Hypotension	n (%)	2 (5.1%)	10 (3.9%)	2 (0.9%)	0.05
Orthostatic Hypotension	n (%)	0	2 (0.8%)	0	

© 2023 Cotter G et al. *JAMA Cardiology.*

System Organ Class<br>Preferred Term	Statistic	Average Dose <50% (N=39)	Average Dose >=50-<90% (N=254)	Average Dose >=90% (N=222)	Trend P-value[#]

Including adverse events with onset date equal to or greater than Day 14 through 90 days post-randomization.
#: Cochran-Armitage trend test. Exact p-value when <5 events in any category

© 2023 Cotter G et al. *JAMA Cardiology.*

eTable 5. Treatment emergent serious adverse events by average percentage optimal dose categories at week 2

System Organ Class<br>Preferred Term	Statistic	Average Dose <50% (N=39)	Average Dose >=50-<90% (N=254)	Average Dose >=90% (N=222)	Trend P-value[#]
Any Serious Adverse Event	n (%)	9 (23.1%)	37 (14.6%)	21 (9.5%)	0.012
Blood And Lymphatic System Disorders	n (%)	0	0	1 (0.5%)	
Febrile Neutropenia	n (%)	0	0	1 (0.5%)	
Cardiac Disorders	n (%)	5 (12.8%)	24 (9.4%)	12 (5.4%)	0.05
Acute Coronary Syndrome	n (%)	0	1 (0.4%)	0	
Acute Myocardial Infarction	n (%)	1 (2.6%)	1 (0.4%)	1 (0.5%)	0.37
Atrial Fibrillation	n (%)	0	1 (0.4%)	0	
Atrioventricular Block Complete	n (%)	1 (2.6%)	0	0	
Cardiac Failure	n (%)	2 (5.1%)	21 (8.3%)	10 (4.5%)	0.31
Cardiac Failure Chronic	n (%)	0	0	1 (0.5%)	
Myocardial Infarction	n (%)	0	0	1 (0.5%)	
Ventricular Tachycardia	n (%)	1 (2.6%)	0	0	
Gastrointestinal Disorders	n (%)	0	1 (0.4%)	1 (0.5%)	
Abdominal Pain	n (%)	0	1 (0.4%)	0	
Abdominal Pain Upper	n (%)	0	0	1 (0.5%)	
Diarrhoea	n (%)	0	0	1 (0.5%)	
General Disorders And Administration Site Conditions	n (%)	1 (2.6%)	2 (0.8%)	1 (0.5%)	
Sudden Death	n (%)	1 (2.6%)	2 (0.8%)	1 (0.5%)	
Hepatobiliary Disorders	n (%)	0	0	1 (0.5%)	
Hepatitis Acute	n (%)	0	0	1 (0.5%)	
Infections And Infestations	n (%)	3 (7.7%)	6 (2.4%)	3 (1.4%)	0.06

© 2023 Cotter G et al. *JAMA Cardiology.*

System Organ Class<br>Preferred Term	Statistic	Average Dose <50% (N=39)	Average Dose >=50-<90% (N=254)	Average Dose >=90% (N=222)	Trend P-value[#]
Bronchitis	n (%)	0	1 (0.4%)	0	
Bursitis Infective	n (%)	0	1 (0.4%)	0	
Corona Virus Infection	n (%)	0	1 (0.4%)	2 (0.9%)	
Gastroenteritis	n (%)	0	1 (0.4%)	0	
Lower Respiratory Tract Infection	n (%)	1 (2.6%)	0	0	
Pneumonia	n (%)	2 (5.1%)	2 (0.8%)	0	
Pneumonia Viral	n (%)	0	0	1 (0.5%)	
Pyelonephritis	n (%)	0	0	1 (0.5%)	
Urinary Tract Infection	n (%)	0	1 (0.4%)	0	
Injury, Poisoning And Procedural Complications	n (%)	0	1 (0.4%)	0	
Fall	n (%)	0	1 (0.4%)	0	
Investigations	n (%)	1 (2.6%)	0	0	
Blood Creatinine Increased	n (%)	1 (2.6%)	0	0	
Metabolism And Nutrition Disorders	n (%)	1 (2.6%)	0	0	
Hyperkalaemia	n (%)	1 (2.6%)	0	0	
Hypoglycaemia	n (%)	1 (2.6%)	0	0	
Musculoskeletal And Connective Tissue Disorders	n (%)	0	0	1 (0.5%)	
Osteochondrosis	n (%)	0	0	1 (0.5%)	
Nervous System Disorders	n (%)	0	3 (1.2%)	1 (0.5%)	
Cerebrovascular Accident	n (%)	0	2 (0.8%)	1 (0.5%)	
Hemianopia Homonymous	n (%)	0	1 (0.4%)	0	
Renal And Urinary Disorders	n (%)	2 (5.1%)	0	1 (0.5%)	0.07
Chronic Kidney Disease	n (%)	1 (2.6%)	0	0	
Renal Impairment	n (%)	1 (2.6%)	0	1 (0.5%)	
Respiratory, Thoracic And Mediastinal Disorders	n (%)	0	1 (0.4%)	1 (0.5%)	

© 2023 Cotter G et al. *JAMA Cardiology.*

System Organ Class<br>Preferred Term	Statistic	Average Dose <50% (N=39)	Average Dose >=50-<90% (N=254)	Average Dose >=90% (N=222)	Trend P-value[#]
Pulmonary Embolism	n (%)	0	0	1 (0.5%)	
Pulmonary Oedema	n (%)	0	1 (0.4%)	0	
Surgical And Medical Procedures	n (%)	0	1 (0.4%)	1 (0.5%)	
Chemotherapy	n (%)	0	0	1 (0.5%)	
High Frequency Ablation	n (%)	0	1 (0.4%)	0	

Including serious adverse events with onset date equal to or greater than Day 14 through 90 days post-randomization.
#: Cochran-Armitage trend test. Exact p-value when <5 events in any category

© 2023 Cotter G et al. *JAMA Cardiology.*

eFigure 1. Change in NT-proBNP by average percentage optimal dose group at Week 2- High Intensity Care Subjects

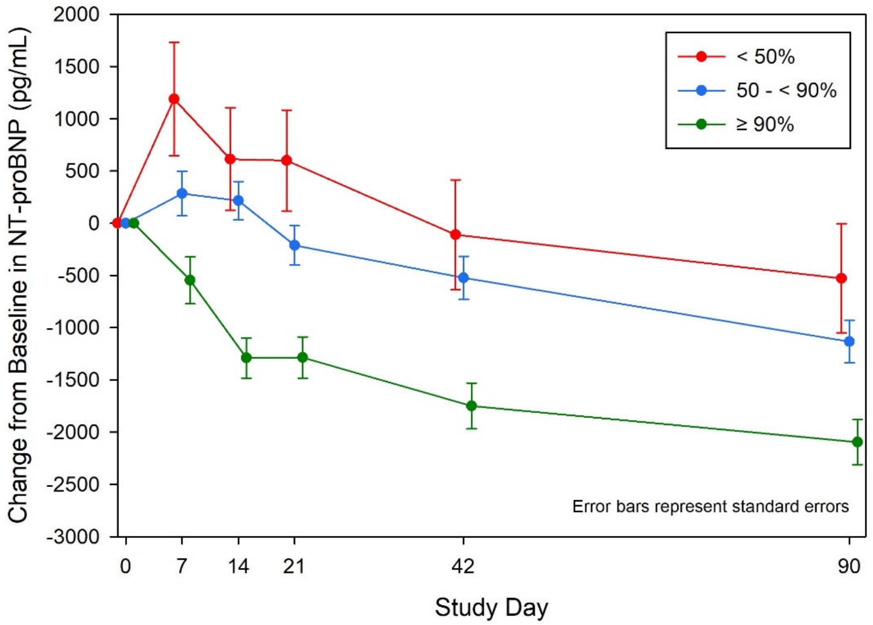

eFigure 2. Change in NT-proBNP by average percentage optimal dose group at Week 2 (or Post-Rand for Usual Care)- All Subjects

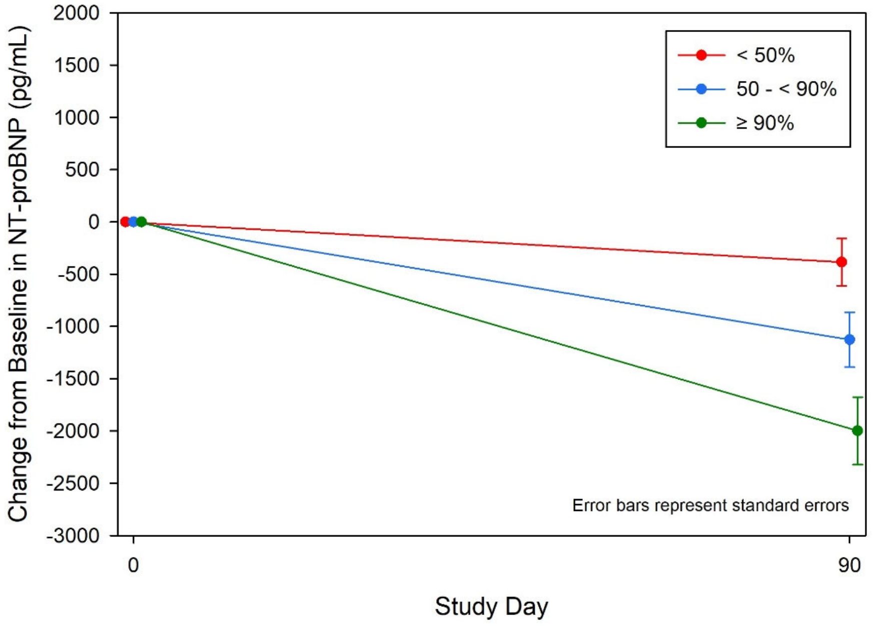

© 2023 Cotter G et al. *JAMA Cardiology.*

eFigure 3. Change in Potassium by average percentage optimal dose group at Week 2- High Intensity Care Subjects

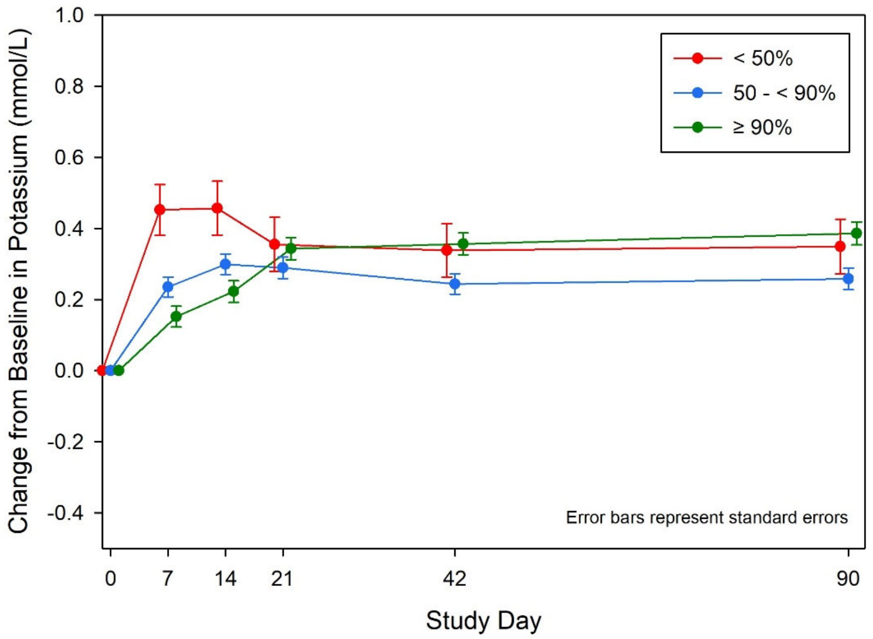

eFigure 4. Change in Potassium by average percentage optimal dose group at Week 2 (or Post-Rand for Usual Care)- All Subjects

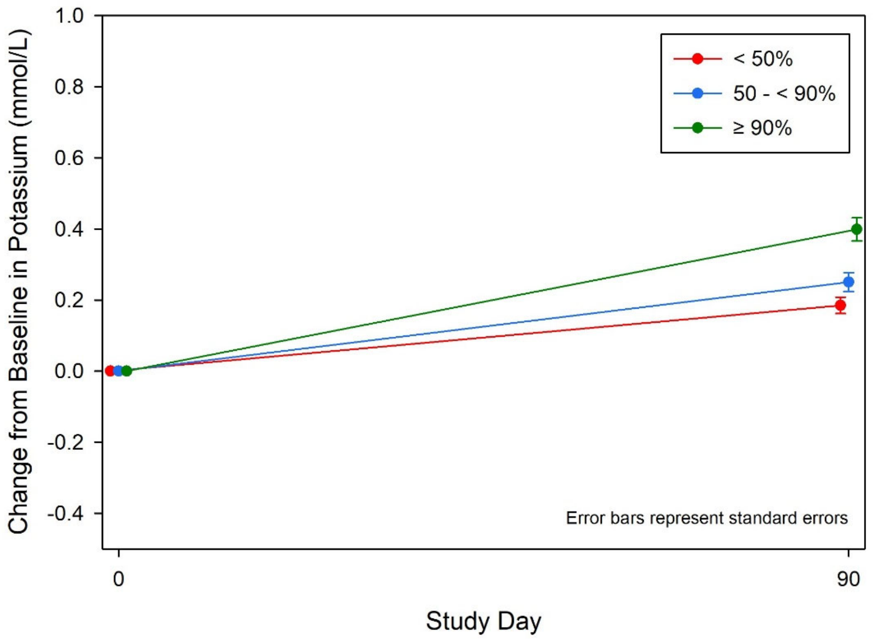

© 2023 Cotter G et al. *JAMA Cardiology.*

eFigure 5. Change in Creatinine by average percentage optimal dose group at Week 2- High Intensity Care Subjects

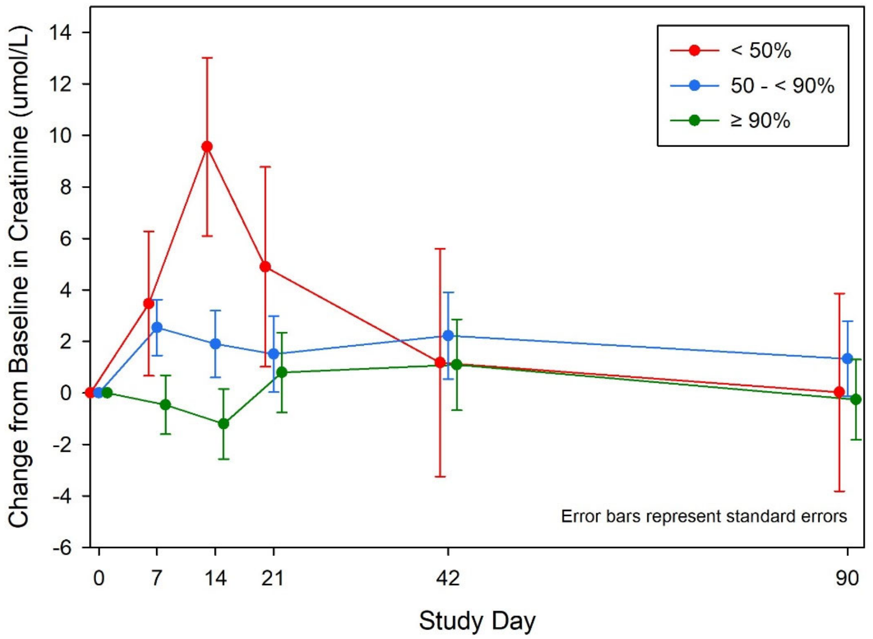

eFigure 6. Change in Creatinine by average percentage optimal dose group at Week 2 (or Post-Rand for Usual Care)- All Subjects

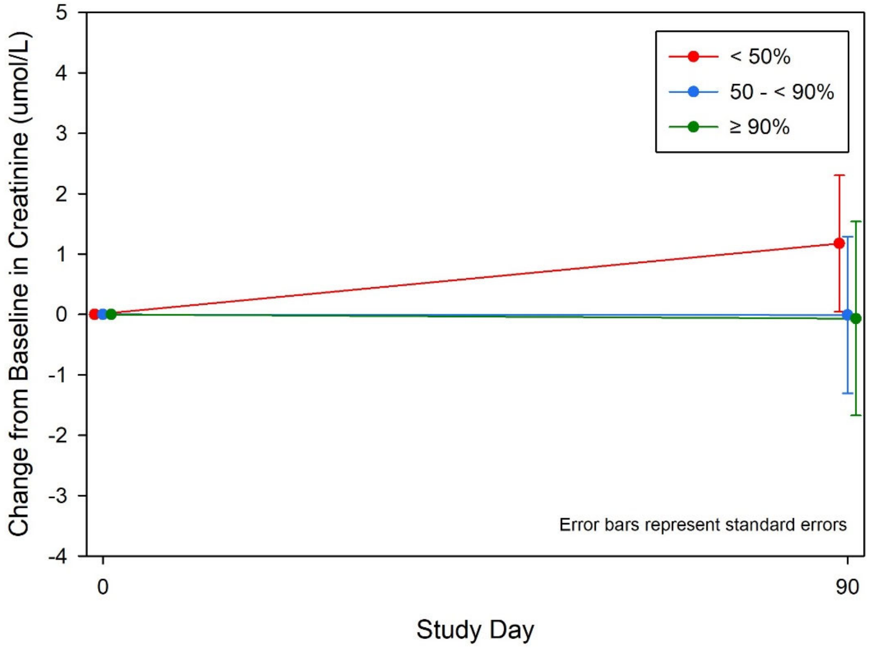

© 2023 Cotter G et al. *JAMA Cardiology.*

eFigure 7. Change in Hemoglobin by average percentage optimal dose group at Week 2- High Intensity Care Subjects

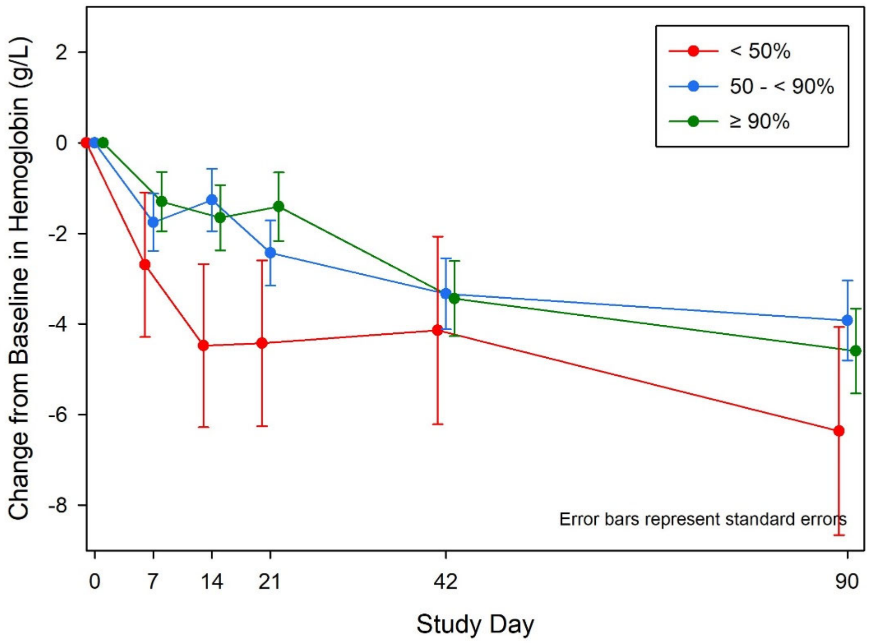

eFigure 8. Change in Hemoglobin by average percentage optimal dose group at Week 2 (or Post-Rand for Usual Care)- All Subjects

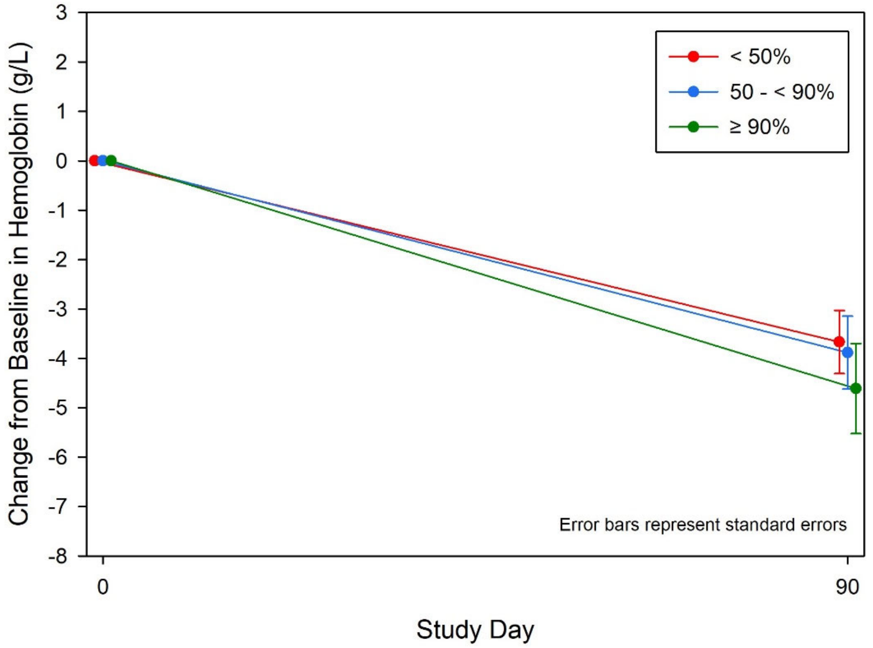

© 2023 Cotter G et al. *JAMA Cardiology.*

eFigure 9. Change in eGFR by average percentage optimal dose group at Week 2- High Intensity Care Subjects

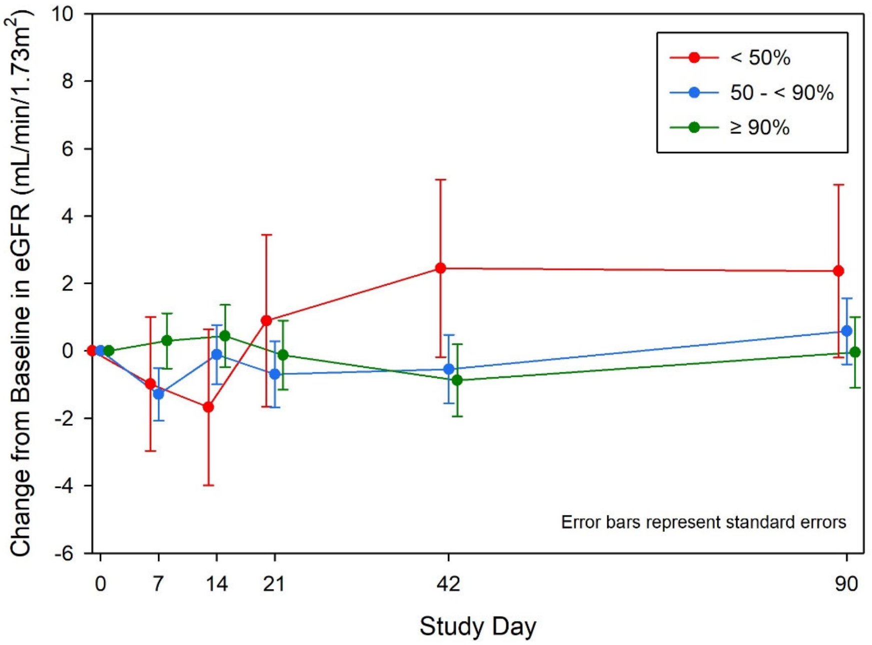

eFigure 10. Change in eGFR by average percentage optimal dose group at Week 2 (or Post-Rand for Usual Care)- All Subjects

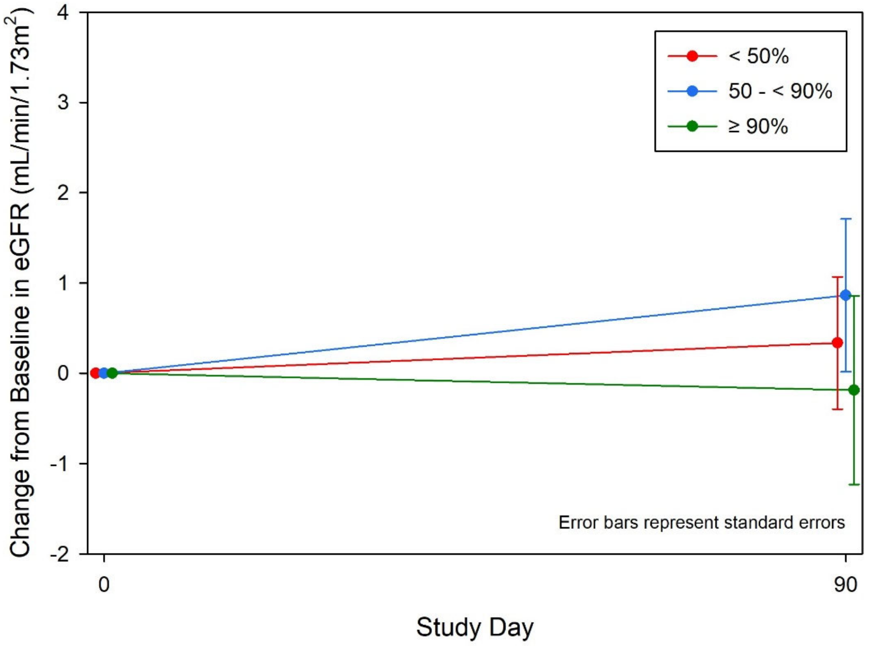

© 2023 Cotter G et al. *JAMA Cardiology.*

eFigure 11. Kaplan-Meier curves for all-cause mortality or heart failure readmission through day 180 by average percentage optimal dose category at Week 2 – High Intensity Care Subjects

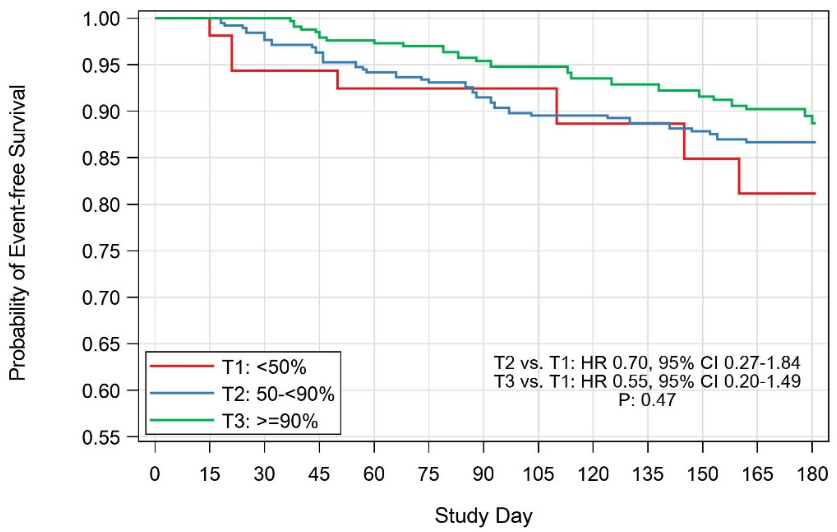

No. at risk

	0	15	30	45	60	75	90	105	120	135	150	165	180
T1: <50%	34	34	32	32	31	31	31	31	30	30	29	28	26
T2: 50-<90%	234	234	230	223	213	211	206	198	197	193	188	182	167
T3: >=90%	214	214	211	202	197	192	187	184	182	179	174	168	147

eFigure 12. Kaplan-Meier curves for all-cause mortality or heart failure readmission through day 180 by average percentage optimal dose category at Week 2 – All Subjects

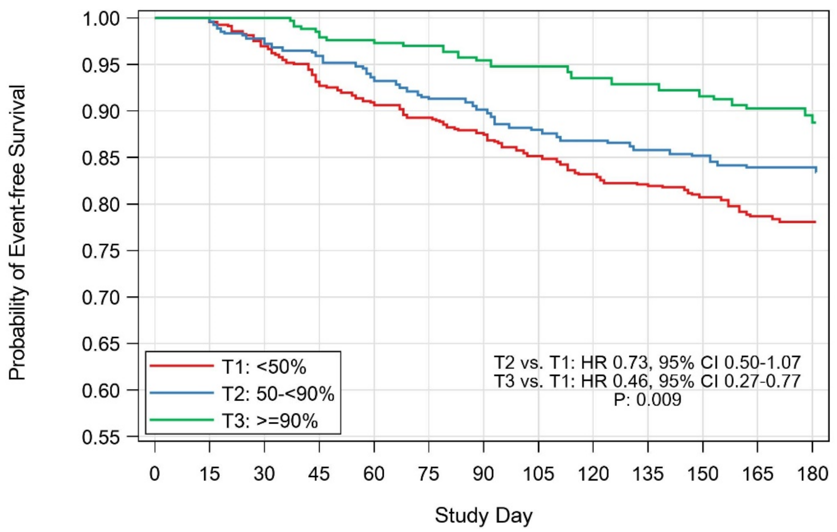

No. at risk

	0	15	30	45	60	75	90	105	120	135	150	165	180
T1: <50%	444	444	426	408	396	384	372	359	350	344	338	327	310
T2: 50-<90%	317	317	309	300	286	280	274	263	257	251	244	235	211
T3: >=90%	215	215	212	203	198	193	188	185	183	180	175	169	148

© 2023 Cotter G et al. *JAMA Cardiology.*

eFigure 13. Kaplan-Meier curves for all-cause mortality through day 180 by average optimal percentage dose category at Week 2 – High Intensity Care Subjects

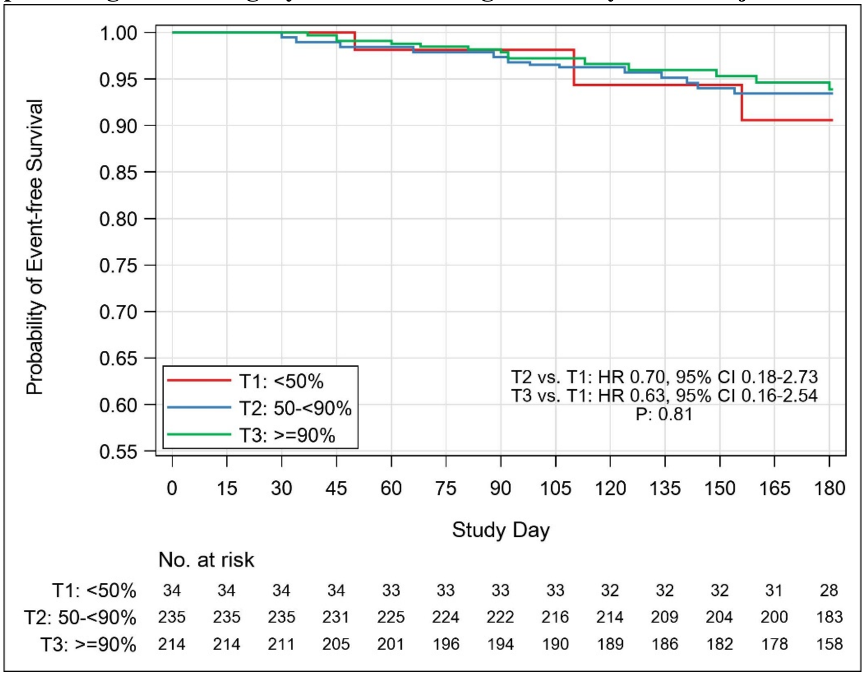

eFigure 14. Kaplan-Meier curves for all-cause mortality through day 180 by average percentage optimal dose category at Week 2 (or Post-Rand for Usual Care) – All Subjects

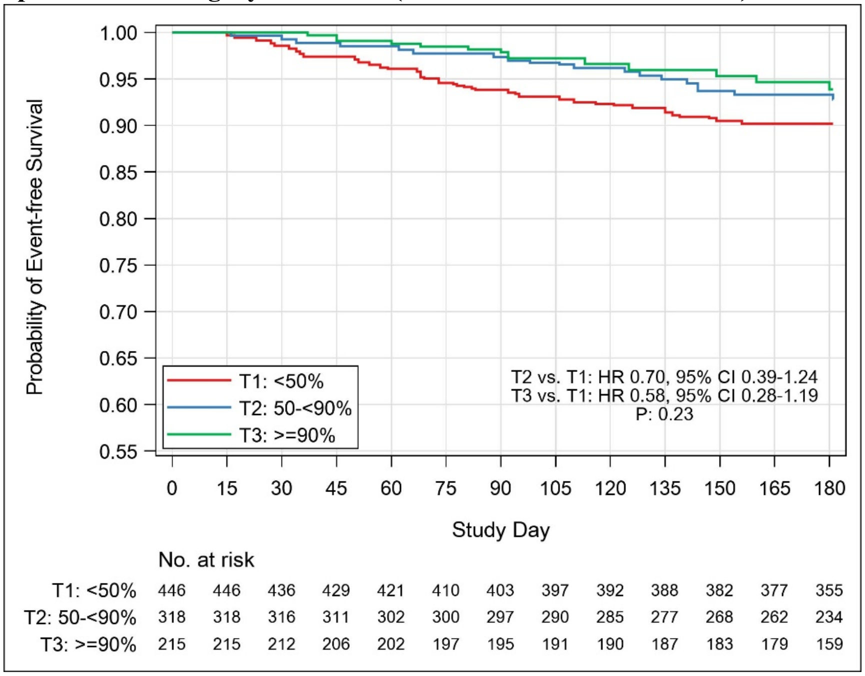

© 2023 Cotter G et al. *JAMA Cardiology.*

eFigure 15. Relative hazard of all-cause mortality or heart failure readmission through day 180 by average percentage optimal dose at week 2 in HIC patients

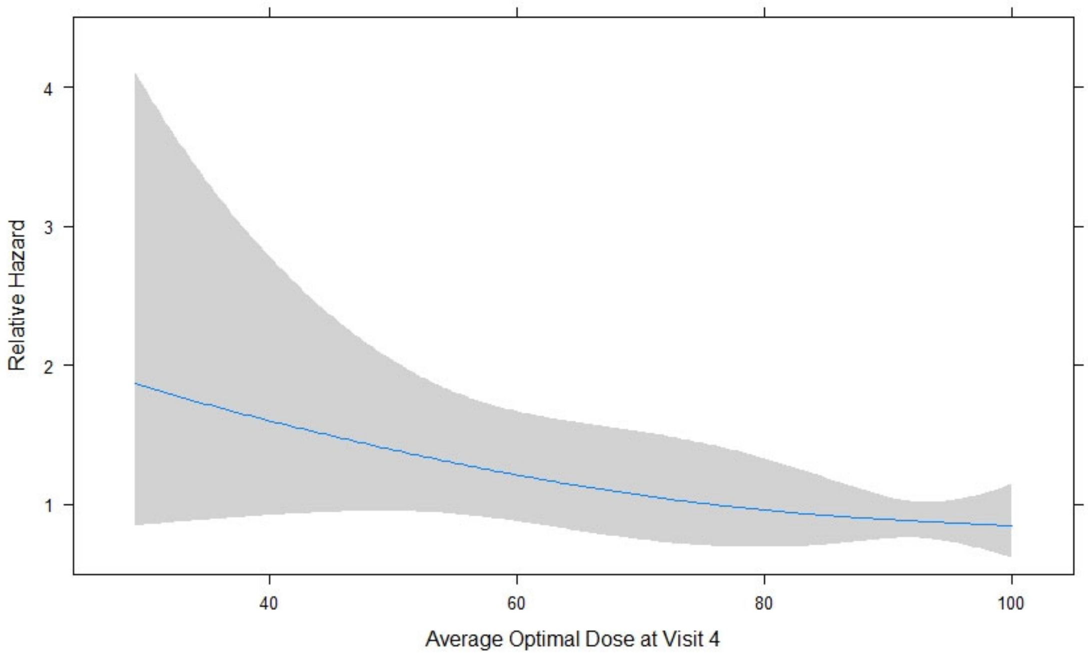

© 2023 Cotter G et al. *JAMA Cardiology.*

eFigure 16. Relative hazard of all-cause mortality or heart failure readmission through day 180 by average percentage optimal dose at week 2 in all subjects

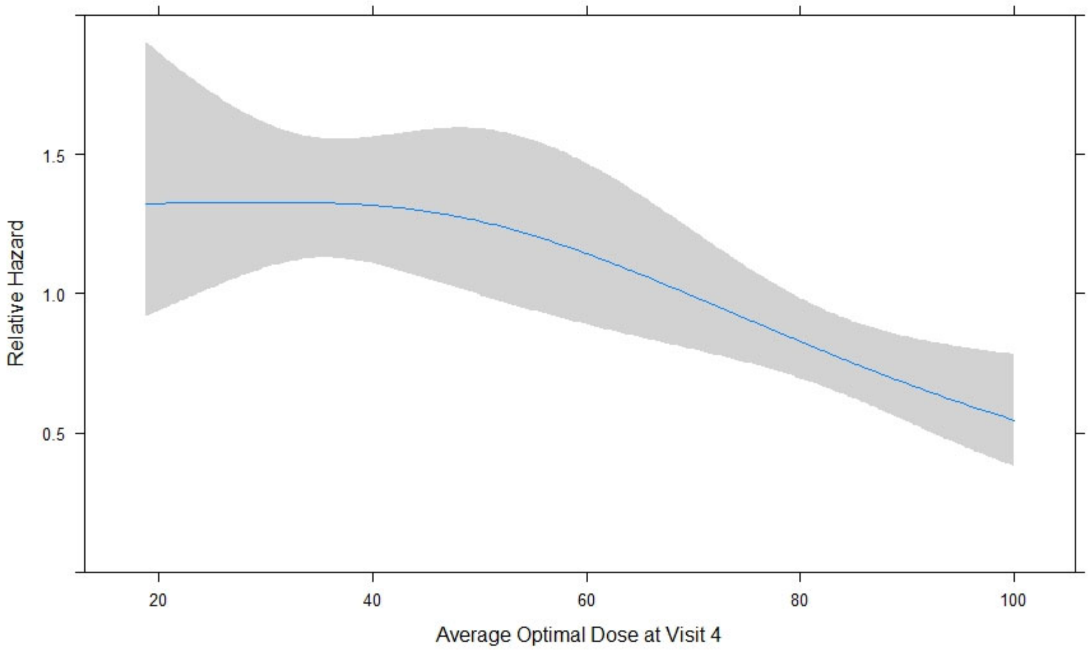

© 2023 Cotter G et al. *JAMA Cardiology.*

eFigure 17. Relative hazard of all-cause mortality through day 180 by average percentage optimal dose at week 2 in HIC patients.

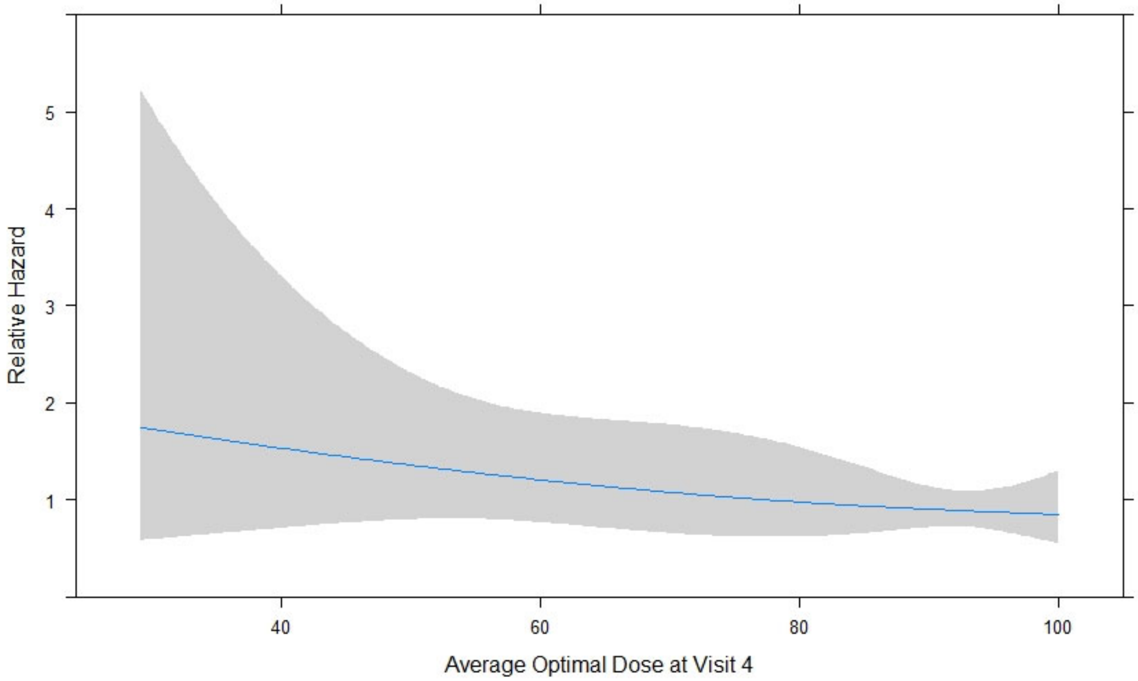

© 2023 Cotter G et al. *JAMA Cardiology.*

eFigure 18. Relative hazard of all-cause mortality through day 180 by average percentage optimal dose at week 2 in all patients

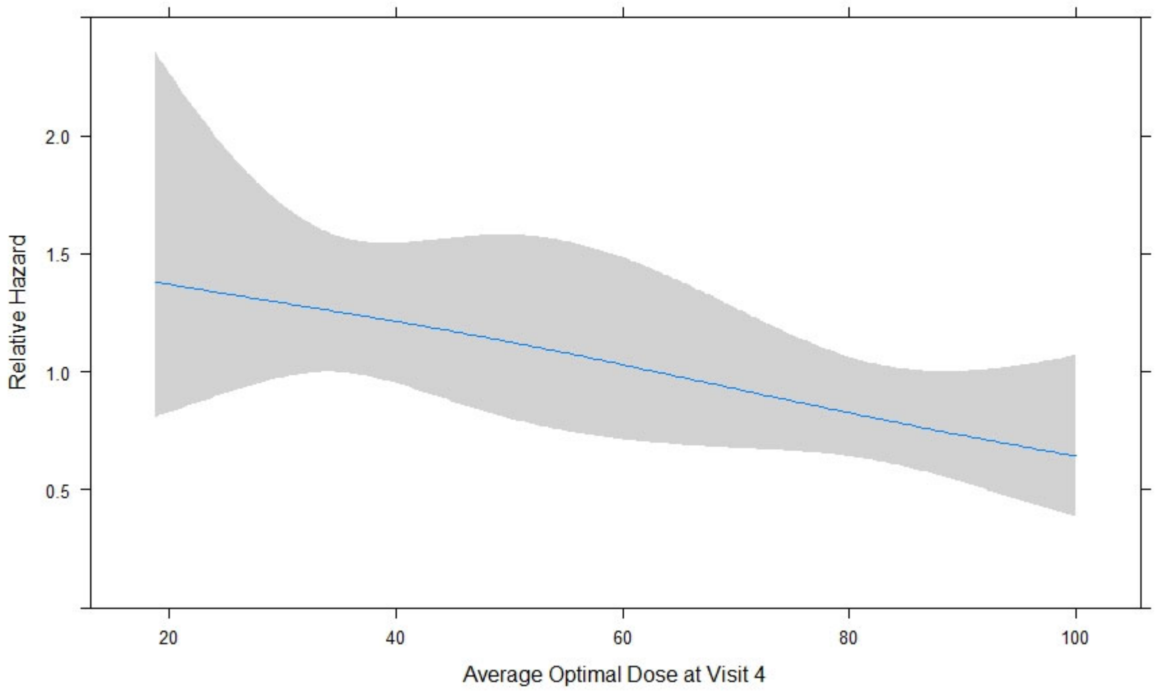

© 2023 Cotter G et al. *JAMA Cardiology.*

eFigure 19. Change from baseline in EQ-VAS to day 90 by average percentage optimal dose at week 2 in HIC patients

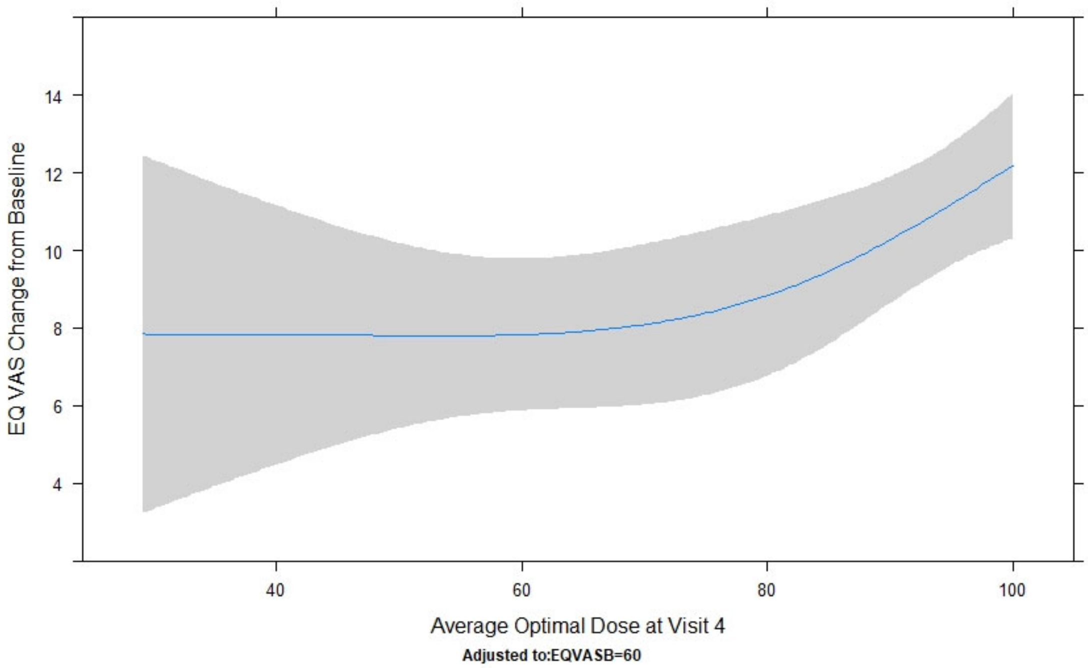

eFigure 20. Bubble Plot of Percent Optimal Dose of RASi by Beta-Blockers at Week 2

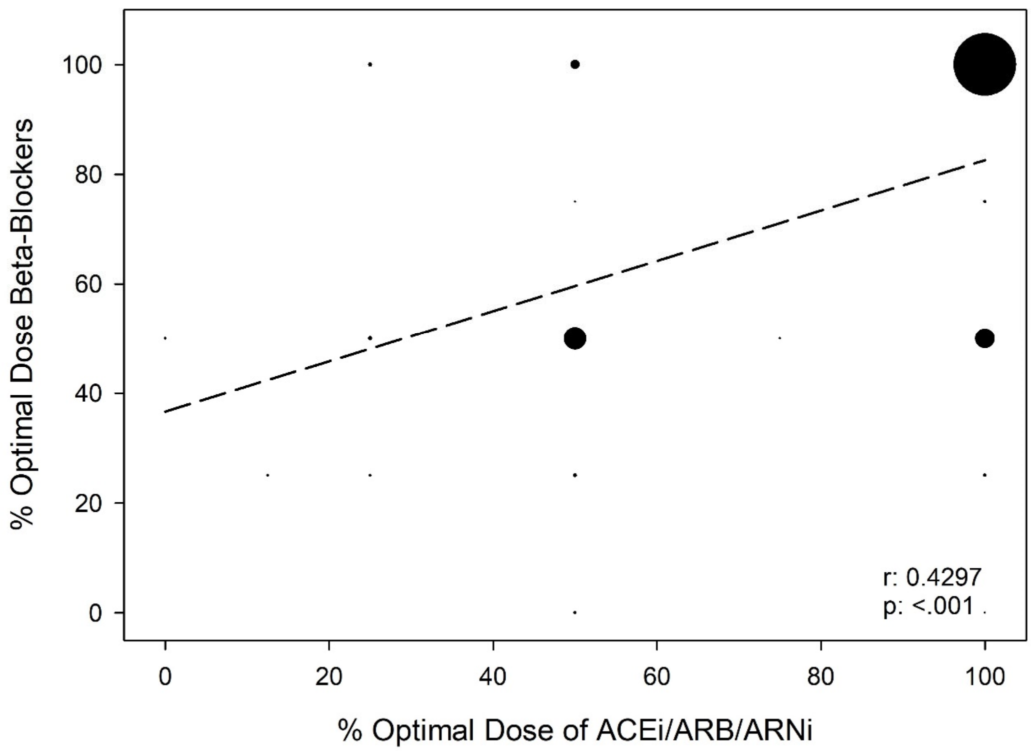

© 2023 Cotter G et al. *JAMA Cardiology.*

eFigure 21. Bubble Plot of Percent Optimal Dose of RASi by MRA at Week 2

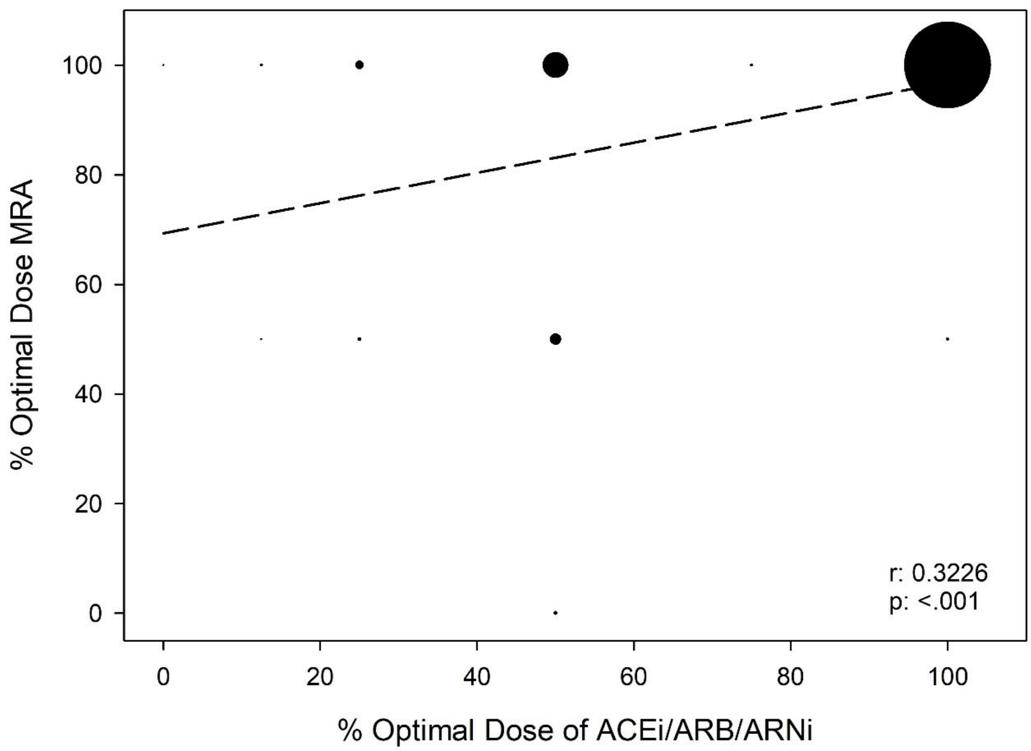

© 2023 Cotter G et al. *JAMA Cardiology.*

eFigure 22. Bubble Plot of Percent Optimal Dose of β-Blockers by MRA at Week 2

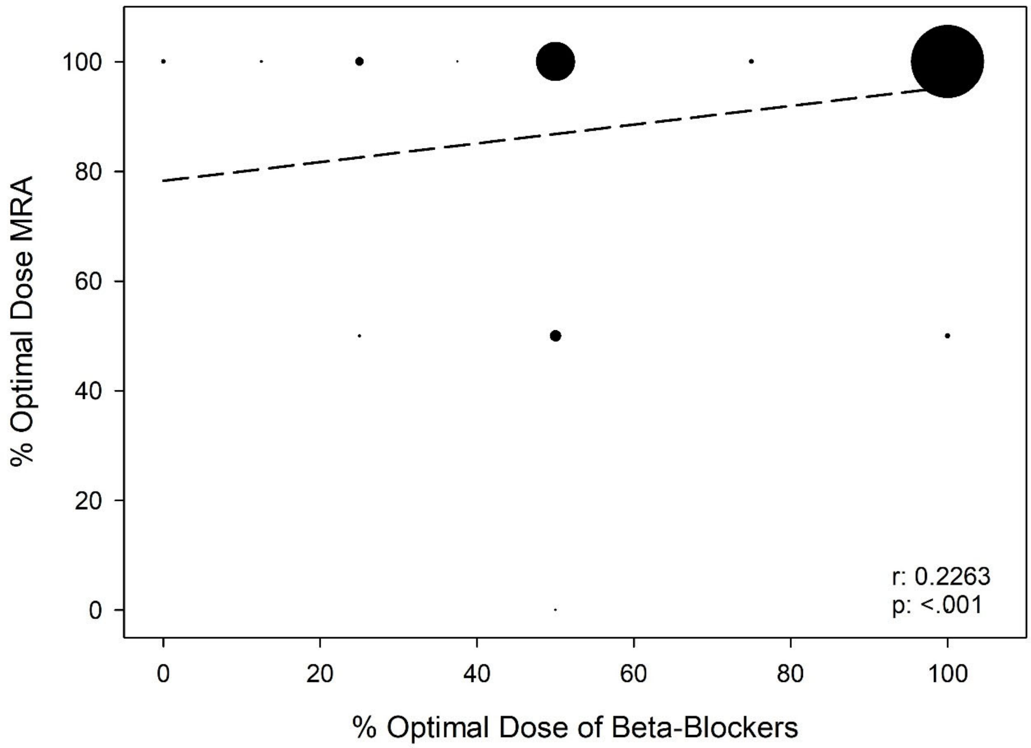

© 2023 Cotter G et al. *JAMA Cardiology.*
