# Supplementary material for: Optimization of Evidence-Based Heart Failure Medications After an Acute Heart Failure Admission: A Secondary Analysis of the STRONG-HF Randomized Clinical Trial
Source: JAMA Cardiol. 2023 Dec 27;9(2):114–24. doi: 10.1001/jamacardio.2023.4553 (PMC10753435; doi:10.1001/jamacardio.2023.4553)
Supplement: Supplement 2. — Data Sharing Statement. [file jamacardiol-e234553-s002.pdf]

Data Sharing Statement

Cotter. Optimization of Evidence-Based Heart Failure Medications After an Acute Heart Failure Admission. *JAMA Cardiol*. Published December 27, 2023. doi:10.1001/jamacardio.2023.4553

Data
Data available: Yes
Data types: Deidentified participant data
How to access data: Individual participant data required to reach aims in an approved proposal, after de-identification, will be made available to investigators whose proposed use of the data has been approved by the study's Executive Committee. Proposals may be submitted up to 36 months after Article publication and should be directed to alexandre.mebazaa@aphp.fr.
When available: With publication

Supporting Documents
Document types: None

Additional Information
Who can access the data: The data will be made available to investigators whose proposed use of the data has been approved by the study's Executive Committee.
Types of analyses: The data will be made available for analyses required to reach aims in an approved proposal.
Mechanisms of data availability: The data will be made available after approval of a proposal, after de-identification. Proposals may be submitted up to 36 months after Article publication and should be directed to alexandre.mebazaa@aphp.fr.
